# In Vivo Intelligent Fluorescence Endo‐Microscopy by Varifocal Meta‐Device and Deep Learning

**DOI:** 10.1002/advs.202307837

**Published:** 2024-03-15

**Authors:** Yu‐Hsin Chia, Wei‐Hao Liao, Sunil Vyas, Cheng Hung Chu, Takeshi Yamaguchi, Xiaoyuan Liu, Takuo Tanaka, Yi‐You Huang, Mu Ku Chen, Wen‐Shiang Chen, Din Ping Tsai, Yuan Luo

**Affiliations:** ^1^ Department of Biomedical Engineering National Taiwan University Taipei 10051 Taiwan; ^2^ Institute of Medical Device and Imaging National Taiwan University Taipei 10051 Taiwan; ^3^ Department of Physical Medicine and Rehabilitation National Taiwan University Hospital & National Taiwan University College of Medicine Taipei 10051 Taiwan; ^4^ YongLin Institute of Health National Taiwan University Taipei 10087 Taiwan; ^5^ Innovative Photon Manipulation Research Team RIKEN Center for Advanced Photonics Saitama 351‐0198 Japan; ^6^ Department of Electrical Engineering City University of Hong Kong Kowloon 999077 Hong Kong, China; ^7^ Department of Biomedical Engineering National Taiwan University Hospital Taipei 10051 Taiwan; ^8^ Centre for Biosystems, Neuroscience and Nanotechnology City University of Hong Kong Kowloon 999077 Hong Kong, China; ^9^ The State Key Laboratory of Terahertz and Millimeter Waves City University of Hong Kong Kowloon 999077 Hong Kong, China; ^10^ Institute of Biomedical Engineering and Nanomedicine National Health Research Institutes Miaoli 35053 Taiwan; ^11^ Molecular Imaging Center National Taiwan University Taipei 10672 Taiwan; ^12^ Program for Precision Health and Intelligent Medicine National Taiwan University Taipei 106319 Taiwan

**Keywords:** deep learning, endoscopy, HiLo fluorescence imaging, metalens, optical sectioning, telecentric configuration, three‐dimensional imaging

## Abstract

Endo‐microscopy is crucial for real‐time 3D visualization of internal tissues and subcellular structures. Conventional methods rely on axial movement of optical components for precise focus adjustment, limiting miniaturization and complicating procedures. Meta‐device, composed of artificial nanostructures, is an emerging optical flat device that can freely manipulate the phase and amplitude of light. Here, an intelligent fluorescence endo‐microscope is developed based on varifocal meta‐lens and deep learning (DL). The breakthrough enables in vivo 3D imaging of mouse brains, where varifocal meta‐lens focal length adjusts through relative rotation angle. The system offers key advantages such as invariant magnification, a large field‐of‐view, and optical sectioning at a maximum focal length tuning range of ≈2 mm with 3 µm lateral resolution. Using a DL network, image acquisition time and system complexity are significantly reduced, and in vivo high‐resolution brain images of detailed vessels and surrounding perivascular space are clearly observed within 0.1 s (≈50 times faster). The approach will benefit various surgical procedures, such as gastrointestinal biopsies, neural imaging, brain surgery, etc.

## Graphical abstract

Intelligent fluorescence endo‐microscopy demonstrates the invariant magnification, large field‐of‐view, and optical sectioning in vivo 3D imaging of mouse brains by the varifocal metalens and deep learning (DL). This study provides a pathway for the next‐generation endo‐microscopy and has excellent potential for the applications of future rapid optical biopsies, such as gastrointestinal biopsies, neural imaging, brain surgery, etc.

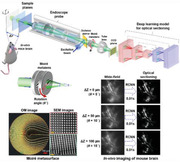

## 1 Introduction

Endo‐microscopy is a rapidly evolving field for the optical visualization of internal organs under minimally invasive conditions. It provides detailed fine features of various tissues and subcellular structures for optical biopsy, which has transformed the diagnosis and treatment of numerous medical conditions. By leveraging advanced optics and high‐speed image acquisition, wide‐field optical endo‐microscopies have emerged as a powerful imaging technique to obtain broad field‐of‐view (FOV) and 3D images of various organs.^[^ ^1^, ^2^ ^]^ Despite its advantages, the lack of optical sectioning capability leads to it facing the challenge of image volumetric tissue conditions due to strong out‐of‐focus background noise.^[^ ^3^, ^4^, ^5^, ^6^ ^]^ Confocal endo‐microscopy is the most widely adopted imaging method that excels in providing high spatial resolution and exceptional out‐of‐focus background rejection.^[^ ^7^, ^8^, ^9^, ^10^ ^]^ However, point‐by‐point scanning of desired voxels within a target tissue can be time‐consuming and cause high photobleaching, which can limit the amount of data that can be acquired and the quality of the resulting images. Nonetheless, deconvolution methods and computational iterative algorithms can improve image quality by reducing noise, increasing contrast, and enhancing resolution. But they have limitations such as high computation time, the requirement of a precise point spread function, and the inability to overcome the missing cone problem, which can limit their practical utility in certain applications.^[^ ^11^, ^12^, ^13^, ^14^ ^]^

Alternatively, structured illumination enables optical sectioning endo‐microscopy in an enticing wide‐field fashion.^[^ ^15^, ^16^ ^]^ It utilizes grid illumination and phase‐shifting computational post‐processing to acquire optically sectioned images at a single depth. Nonetheless, the phase‐shifting approach mandates the acquisition of at least three‐phase illuminated patterns onto the tissue, followed by a demanding demodulation process. To circumvent these limitations, a comprehensive, structured illumination for HiLo endo‐microscopes has been developed,^[^ ^3^, ^17^ ^]^ exhibiting an exceptional ability to obtain sectioning images efficiently. Furthermore, the utilization of speckle illumination in the HiLo approach can facilitate deep tissue penetration.^[^ ^18^, ^19^, ^20^ ^]^ Even though HiLo endo‐microscopy renders remarkable optical sectioning images, it necessitates axial scanning by moving the endoscope probe or sample, thereby greatly increasing the risk of injuring tissue from external compression. The optically sectioned HiLo images need to be additionally computed from pairwise images.

In general, axial focal positions in endo‐microscopy can be controlled by tunable lenses of different types.^[21^ ^]^ However, conventional zoom lenses, with their composite construction, present difficulties in achieving miniaturization.^[22^ ^]^ Liquid lenses, which rely on tunable curvature, are limited by the effects of gravity and tend to produce aberrations that degrade image quality.^[23^ ^]^ Addressing these challenges, Bernet et al. recently presented a Moiré lens, a paired diffractive optical elements (DOEs) lens, for focus tunability.^[^ ^24^, ^25^, ^26^, ^27^ ^]^ By tuning the mutual angles of complementary phase masks, focal lengths can be continuously varied without axial movement. Conventional DOEs are binary‐phase gratings, suffering from low efficiency and low uniformity.^[28^ ^]^ In addition, the fabrication of DOEs with complex morphology and compact size remains a significant challenge that requires further attention.

The contemporary advent of metasurfaces, as a groundbreaking technique for manipulating light, has given rise to unparalleled solutions for optical system designs.^[29^ ^[^ These ultrathin and planar optical elements possess an exceptional ability to tailor light properties,^[30^ ^]^ thus promising seamless integration into an array of devices.^[^ ^31^, ^32^, ^33^, ^34^, ^35^, ^36^ ^]^ In comparison to conventional refractive optical counterparts, a metalens is able to offer a lightweight, miniaturized, and high degree of freedom lens, which has great potential to be integrated into optical systems.^[^ ^37^, ^38^, ^39^, ^40^, ^41^, ^42^, ^43^, ^44^, ^45^ ^]^

In the present, the exponential advancement in computing power, particularly the remarkable progress in computer and GPU‐related technologies, causes a transformative era for artificial intelligence (AI). AI has surged to the forefront in biomedical imaging, acting as a leading methodology for computer‐assisted interventions,^[46^ ^]^ quantitative phase imaging,^[47^ ^]^ and high‐resolution fluorescence imaging.^[48^ ^]^ With its capacity to transcend hardware restrictions and consolidate the advantages of different optical techniques in imaging, classification, and regression problems,^[^ ^49^, ^50^, ^51^, ^52^, ^53^, ^54^, ^55^, ^56^, ^57^, ^58^ ^]^ AI promises to engender significant improvements in image quality. Integrating metasurfaces with AI represents one of the pioneers in futuristic optical systems. The precise control over light by metasurfaces, coupled with the learning‐based adaptability of AI, creates a synergistic partnership, enabling novel capabilities for high‐resolution imaging, real‐time diagnostics, and intelligent decision‐making in optical‐based applications. Even with the latest endo‐microscopic optical instrument, there is still enormous scope to improve using deep learning (DL) methods.^[46^ ^]^

So far, several works have been reported to utilize metalens in endoscopic systems, which include fiber bundle endoscopy,^[59^ ^]^ optical coherence tomography,^[^ ^60^, ^61^ ^]^ and two‐photon micro‐endoscopy.^[62^ ^]^ Nevertheless, none of the studies can perform in vivo fluorescence optical sectioning images. In this work, a Moiré metalens based DL fluorescence endo‐microscopy capable of observing optically sectioned in vivo images with high resolution is demonstrated. The Moiré metalens is realized by using two complementary dielectric metasurfaces.^[63^ ^]^ With a telecentric configuration,^[64^ ^]^ the micro‐endoscope reaches uniform image magnification, which is critical for 3D biomedical imaging. To empower optical sectioning capability, speckle illumination for HiLo imaging is applied to the endo‐microscope. Furthermore, to reduce imaging acquisition time, the DL method is adopted to achieve HiLo imaging in a single shot. The imaging process time is faster than the traditional HiLo imaging process. The high‐resolution images are obtained by the DL method directly. We have experimentally demonstrated 3D in vivo mouse brain imaging using the Moiré metalens. Our system obtains high‐resolution mouse brain images of both transparent and opaque mouse brains, up to 200 µm and 100 µm in depth, respectively. To the best of our knowledge, our approach is the first report on the implementation of metalens for focus tunable fluorescence endo‐microscopy to achieve a long axial scanning range for deep tissue in vivo imaging. In addition, a DL model to assist in vivo optical sectioning of the mouse brain and analytical expressions for the focus tunability of endoscopic systems are presented. The endo‐microscope has multiple functionality and clinical viability, which augment existing systems in performing rapid in situ volumetric imaging before surgeries. With the aid of the image‐to‐image translation residual convolutional neural network (RCNN) DL model, we have accurately and rapidly performed in vivo optical sectioning images of detailed brain vessels and surrounding perivascular space. The present method can directly help in investigating the functioning of the glymphatic system in live organisms by utilizing a cerebrospinal fluid (CSF) tracer to track its flow through the perivascular spaces into the brain tissue.

**Figure 1:**
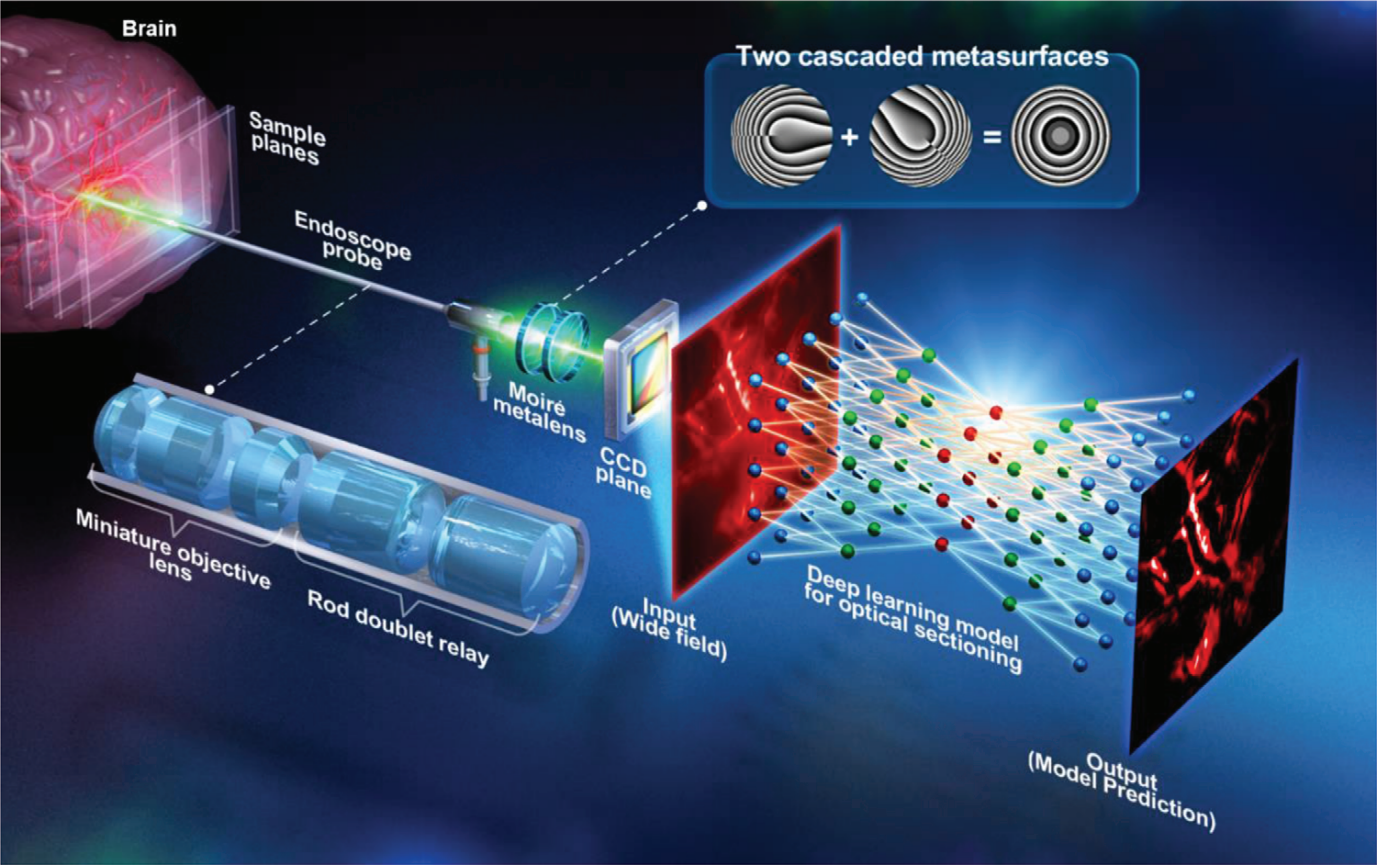
Schematic of the varifocal metalens based intelligent fluorescence endo‐microscopy. A Moiré metalens is positioned at the system's Fourier plane to tune focal points for 3D imaging. Fluorescence images of the in vivo brain at different depths are captured by the endoscope probe, and the RCNN model for the HiLo process achieves optical sectioning imaging in a wide‐field manner within a single shot.

The schematic of the proposed fluorescence optical sectioning endo‐microscopy is shown in **Figure** **1**. A customized rigid endoscopic probe is stretched into and approached to a target brain area, and fluorescence subcellular brain images are captured by the endoscopic probe. We introduce a miniaturized Moiré metalens into the endo‐microscope, which is composed of two phase metasurfaces (highlighted box region in Figure 1) for 3D imaging without any axially moving components in depths. Due to telecentric configuration, system FOV maintains constant during axial scanning by rotating different angles between two metasurfaces. Ex vivo transparent mouse brain tissues are used to demonstrate the fine optical sectioning capability of the endo‐microscope by utilizing the HiLo imaging method with pairwise images (i.e., one uniform illumination and one speckle illumination). In order to expedite the entire processing time of HiLo optical sectioning, we adopt a DL^[^ ^65^ ^]^ RCNN^[^ ^66^, ^67^ ^]^ model algorithm to obtain 3D optical sectioning images of in vivo mouse brains. It only requests one image under uniform illumination, and the well‐trained RCNN model predicts a high‐contrast optical sectioning image within a single shot in real time.

## 2 Result and Discussion

### 2.1 Customized Rigid Endoscope Probe

The customized endoscopic probe is designed and optimized by OpticStudio ray‐tracing software (Zemax, LLC).^[68^ ^]^ The probe is composed of two parts: a miniature objective lens and a rod doublet relay, as shown in Figure 1. The total length of our customized rigid endoscope probe is ≈18 cm. In the first part, the miniature objective lens includes two doublet lenses and one meniscus lens, which are designed based on the Cooke triplet objective lens.^[69^ ^]^ The diameter and numerical aperture (NA) of the miniature objective lens are 8 mm and 0.26, respectively. In the second part, the rod doublet relay consists of a rod relay lens and an achromatic doublet relay. The rod relay lens includes two sets of field lenses and a Hopkins rod relay with an optimized radius of curvature on all surfaces. Compared to a conventional relay system, the Hopkins rod relay incorporates a glass rod that improves light throughput and reduces vignetting.^[70^ ^]^ The achromatic doublet relay consists of two kinds of achromatic doublet lenses (AC254‐50‐A and AC254‐100‐A) capable of decreasing spherical aberration and field curvature.

### 2.2 Design of Moiré Metalens

The Moiré metalens consists of two cascaded metasurfaces,^[^ ^25^, ^26^, ^63^ ^]^ which are made of Gallium Nitride (GaN) dielectric material.^[^ ^35^, ^71^ ^]^ The complex conjugate transmission functions of the two metasurfaces (D1, D2) are

(1) $$D_{1}\left(R,\varphi \right)=e^{\left[i\;\mathrm{round}\;\left(cR^{2}\right)\varphi \right]}$$

(2) $$D_{2}\left(R,\varphi \right)=e^{\left[-i\;\mathrm{round}\;\left(cR^{2}\right)\varphi \right]}$$

where *R* =$\;\sqrt{x^{2}+y^{2}}$, *ϕ* = atan2(*y*,*x*), and *c* = *π*⁄*λf*. *R* denotes the position in the radial coordinate, Φ is the polar angle, and *c* is a constant, which can be controlled by adjusting the focal length (*f*), and operation wavelength (*λ*). The phase profile of the metalens is restricted in the range 0–2π, which is relevant to a Fresnel lens.^[72^ ^]^ The round(⋅) is the function of rounding the input parameter to the nearest integer number^[^ ^25^ ^]^ that can effectively avoid a discontinuity at the region of the polar angle equal to π. In the actual function of our Moiré metalens, the paired metasurfaces need to overlap together. When the second metasurface is rotated with an angle (*θ*) relative to the first one, the transmission function of the Moiré metalens (*D*_Moire_) becomes the following:

(3) $$\begin{array}{cc} D_{\mathrm{Moire}}\left(R,\varphi \right) & =D_{1}\cdot D_{2}=e^{\left[i\;\mathrm{round}\;(cR^{2})\varphi \right]}\cdot e^{\left[-i\;\mathrm{round}\;\left(cR^{2}\right)\left(\varphi -\theta \right)\right]}\\ & =e^{\left[i\;\mathrm{round}\;\left(cR^{2}\right)\theta \right]} \end{array}$$

According to the transmission function of an ideal lens,^[^ ^26^, ^73^ ^]^ the effective focal length of the Moiré metalens can be defined as

(4) $$f_{\mathrm{Moire}}=\frac{\pi }{\lambda \cdot c\cdot \theta }$$

From Equation (4), the focal length (*f*_Moire_) of our Moiré metalenses is inversely proportional to the rotation angle between the paired metasurfaces. The 800 nm‐height GaN nanopillars with various diameters are designed for 2π phase modulation of the designed Moiré phase arrangement, as shown in Section 1 of Supporting Information. The fabrication details of the Moiré metalens can be found in the Experimental Section and Section 2 of Supporting Information.

### 2.3 Design of Telecentric Configuration

The varifocal observation capability of our system is achieved by telecentric configuration has the advantage of providing invariant magnification and contrast for optical imaging.^[64^ ^]^ The ray transfer matrix (i.e., ABCD matrix) is applied to perform telecentric design.^[74^ ^]^ ABCD matrix derivation for telecentric design is discussed in Section 3 and 4 of Supporting Information. Initially, the system adheres to the telecentric design to position the Moiré metalens at the Fourier plane of the micro‐endoscope (Figure 1). Subsequently, our endoscopic system FOV can remain constant during varifocal observation by rotating different angles between two metasurfaces. The detailed verification of invariant magnification property and configuration of telecentric endo‐microscopy combined with Moiré metalens can be found in Figure S3 (Supporting Information), and the maximum focal length tuning range is ≈2 mm with 3 µm lateral resolution. The telecentric characterization of experimentally measured beam profiles with a tuning range of ≈6 mm at three different operation wavelengths of blue (491 nm), green (532 nm), and red (633 nm) can also be found in Section 5 of Supporting Information.

### 2.4 Varifocal Optical Sectioning Endo‐Microscopy

**Figure 2:**
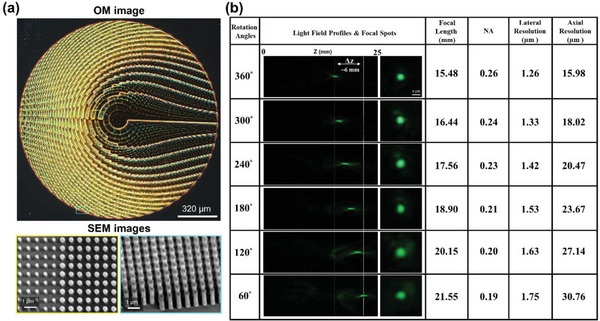
Experimental measurement of varifocal optical sectioning endo‐microscopy. a) The optical microscopy (OM) and scanning electron microscope (SEM) images of our Moiré metasurface (diameter is 1.6 mm, nano cylinder height is 800 nm, and period is 300 nm). b) Telecentric focusing measurement for green wavelength at 532 nm (the rotation angles from 360° to 60° can create the focal length difference Δz ∼ 6 mm). Different NA can generate corresponding lateral and axial resolutions.

Moiré metalens is positioned in the Fourier plane of the system. The Moiré metalens is mounted on an electrically controlled rotation stage (GT45, Dima Inc., maximum rotation speed: 140 RPM with 0.004° maximum rotation accuracy in each step) to tune the focus by changing the relative angle between the paired metasurfaces. To precisely adjust the rotation stage, a data acquisition card (myDAQ, NI Inc.) is used for automation. The Moiré metasurface optical microscopy (OM) and scanning electron microscope (SEM) images are shown in **Figure** **2**a. In the telecentric measurement, we use the green laser (532 nm) as the light source in Figure 2b. The rotation angles of the Moiré metalens are adjusted from 360° to 60° with a step size of 60°, and the corresponding focal length of the endoscope probe can be tuned from ≈15.5 to ≈21.6 mm (Δz = ≈6 mm). The NA of the endoscope probe varies from ≈0.26 to ≈0.19. To quantify the optical sectioning ability of our micro‐endoscope, fluorescently labeled microspheres (Fluoresbrite YG microspheres, 90 µm in diameter, Polysciences Inc.) are sequentially scanned along the axial direction. The detailed HiLo imaging principle and varifocal optical sectioning endo‐microscopy system calibration is described in Sections 6 and 7 of Supporting Information). The 3D reconstruction volume images of mouse brain various perivascular space locations in uniform illumination and HiLo process are shown in Section 7 of Supporting Information. In addition, the ex vivo mouse brain dyed with Alexa Fluor 488 imaging results can be found in Section 8.

### 2.5 Deep Learning for Optical Sectioning Imaging

**Figure 3:**
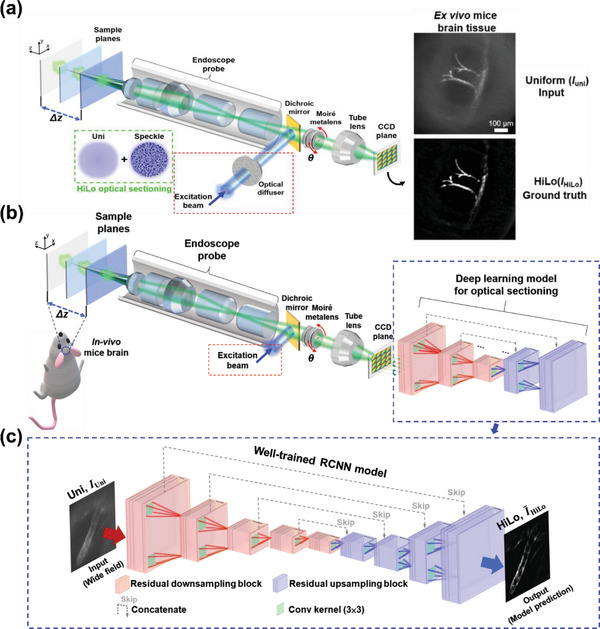
RCNN DL model for optical sectioning endo‐microscopy. a) The experimental setup of the Moiré metalens optical sectioning endo‐microscopy for DL model training and validating datasets. Randomly selected data of five different types of fluorescent samples with 2800 training and 700 validation dataset image pairs (see Section 9 of Supplementary Materials). An optical diffuser is added to the micro‐endoscopic system for HiLo sectioning method to generate speckle illumination (green dash square). b) The proposed RCNN architecture for HiLo optical sectioning. Red square regions represent residual down‐sampling blocks to reduce the dimensionality of image features. Blue square regions represent residual up‐sampling blocks, which bring back low dimensional features equal to the original input size. c) RCNN DL model for optical sectioning endo‐microscopy. In our varifocal optical sectioning endo‐microscopy) the RCNN DL model is used to replace the conventional HiLo method to simplify optical sectioning acquisition process.

The HiLo imaging method is utilized in the endo‐microscope to generate high‐resolution images with fine optical sectioning capacity, as shown in **Figure** **3**a. The experimental setup of the Moiré metalens optical sectioning endo‐microscopy of HiLo imaging is used for obtaining DL model training and validating datasets. Conventionally, HiLo imaging under speckle illumination still requires paired images, one is under uniform illumination (*I*_uni_), and the other is under speckle illumination (*I*_sp_), to obtain an optical sectioning image (i.e.*, I*_HiLo_). For the uniform imaging, the excitation green laser beam (λ = 532 nm, Cobolt Inc.) is reflected by a dichroic mirror (DMLP567R, Thorlabs Inc.), located between the endoscope probe and Moiré metalens, and propagates through the endoscope probe to directly illuminate the specimen. The red emission light generated by the fluorescently labeled specimen is then collected by the endoscope probe and displayed onto the CCD (GE1650, Prosilica Inc.) to generate the uniform imaging. For the speckle imaging, the excitation laser beam passes through an optical diffuser to produce randomly distributed speckle patterns along the axial direction to generate the speckle illumination for the HiLo imaging process. Randomly selected data of five different types of fluorescent samples with 2800 training and 700 validation dataset image pairs are shown in Section 9 of Supporting Information. The entire process of conventional HiLo is time‐consuming and makes the system too complicated. To overcome these limitations, the micro‐endoscope utilizes a RCNN DL model for HiLo sectioning images. Figure 3b shows the configuration of the RCNN DL model for the HiLo optical sectioning imaging technique. In the proposed RCNN DL model, the input is uniformly illuminated *I*_uni_ obtained by the endo‐microscopy, and a well‐trained RCNN model transfers the *I*_uni_ images into optically sectioned $\tilde{I}$_HiLo_ images, as shown in Figure 3c. A more detailed discussion of the proposed RCNN architecture can be found in Section 10 of Supporting Information. Using the RCNN DL model, the optical diffuser can be removed from the endo‐microscope to reduce the setup complexity, as shown in the red dashed boxes of Figure 3a,b. In addition, the acquisition speed of optical sectioning images is significantly enhanced up to 0.1 s (≈50 times faster). The 3500 image pairs of *I*_uni_ and *I*_HiLo_ are composed of five different types of fluorescent samples, including ex vivo mouse brain tissue, fluorescent beads, corn stem, lily anther, and basswood stem, captured by our endo‐microscopy for both training and validation datasets of the RCNN model. The 2800 image pairs (80%) and 700 image pairs (20%) are used for training and validation, respectively, as shown in Figure S13 (Supporting Information). The validation datasets do not exist in the training dataset. Image data of five different types provide different features to the model, such as slender, hollow, and circular structures, to expand the model's versatility. Depending on the supervised learning method, the *I*_uni_ images are set as the input, and the corresponding *I*_HiLo_ images are the ground truth. In order to intuitively operate the model, input, and ground truth images are scaled to the same size (256×256 pixels).

**Figure 4:**
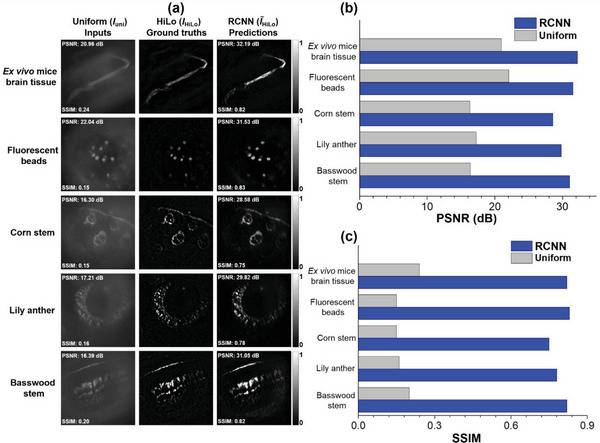
Comparison of inputs, ground truths, and predictions from validation dataset. a) Resultant images of five different types of fluorescent samples that include ex vivo mouse brain tissues, fluorescent beads, corn stem, lily anther and basswood stem taken by validation dataset. b) The PSNR comparison analysis bar chart for inputs and model predictions. c) The SSIM comparison analysis bar chart for inputs and model predictions.

To evaluate the prediction results of the RCNN model, *I*_uni_, *I*_HiLo_, and $\tilde{I}$_HiLo_ is compared in **Figure** **4**. Figure 4a shows the resultant images of five fluorescent samples from the validation dataset, and the resultant images of the training dataset are discussed in Section 12 of Supporting Information. The absolute error map is utilized to visualize their difference, as shown in Supporting Information Section 11 Figure S16.

In order to quantitatively evaluate the well‐trained RCNN model, both peak signal‐to‐noise ratio (PSNR) and structural similarity index (SSIM) evaluation metrics are measured. The PSNR indicates the quality of the images, and the SSIM can quantify the similarity of the model‐predicted and ground truth images^[^ ^75^ ^]^ (the detailed definitions of PSNR and SSIM are described in Section 13 of Supporting Information). In **Figure** **4**a, the PSNR and SSIM of each *I*_uni_ and $\tilde{I}$_HiLo_ image are shown in the corner. Moreover, the comparison bar chart for the PSNR and SSIM is shown in Figure 4b,c, respectively. Compared to the input images, the RCNN predicted images can significantly improve both evaluation metrics for all five different types of fluorescent samples (the average PSNR and SSIM values for *I*_uni_ and $\tilde{I}$_HiLo_ from the validation dataset is described in Section 13 of Supporting Information). To verify the versatility and stability of the RCNN model, ex vivo mouse brain tissues without the RapiClear transparent process are fluorescently labeled with Alexa Fluor 555 for the testing dataset, which does not exist in both training and validation datasets (in Section 14 of Supporting Information).

### 2.6 In Vivo Imaging of Mouse Brain for Preclinical Usage

**Figure 5:**
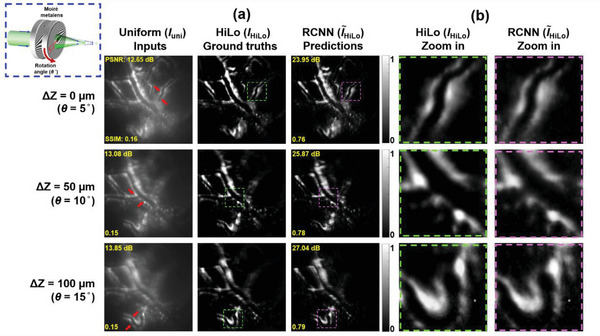
In vivo fluorescence images of mouse brain. a) In vivo images of wide‐field, ground truth, and model predictions at three different depths (the corner dashed box show the rotation of Moiré metalens for the corresponding focal plane, and the arrows in *I*_uni_ point out the focused area). b) Zoom in results corresponding to the dash square region in (a), the left column (green dash square), and the right column (purple dash square) show the *I*_HiLo_ and $\tilde{I}$_HiLo_ zoom in images, respectively.

To evaluate our endo‐microscopy for 3D live fluorescence imaging applications, we further perform in vivo images of a mouse brain (image depth: 100 µm), which is labeled with a fluorescence tracer (Alexa Fluor 555). The detailed surgical and fluorescently labeled preparation procedure of the in vivo mouse brain is described in the Section of Materials and Methods. While imaging, the anesthetized mice are fixed onto a customized stereotaxic frame (Stoelting Inc.), and the surgical incision of the brain can face the endoscopic probe. The stereotaxic frame is positioned onto a three‐axis linear stage (TSD‐652S‐M6 & TSD‐653L‐M6, OptoSigma Inc.) for fine alignment. Resultant images of the in vivo mouse brain are shown in **Figure** **5**. The green excitation beam (λ = 532 nm) is used to illuminate the brain, and in vivo fluorescence images in red (λ = ∼600 nm) are observed. Figure 5a shows three in vivo images (*I*_uni_, *I*_HiLo_, and $\tilde{I}$_HiLo_) of the parenchymal brain at different axial positions by rotating the Moiré metalens with the corresponding angles of *θ* = 5, 10°, and 15°. In Figure 5a, the $\tilde{I}$_HiLo_ images using the well‐trained RCNN model provide fine optical sectioning images, which are comparable to the ground truth (i.e.*, I*_HiLo_). The background inside vessels (i.e., the black tube area in Figure 5a) and the tracer of the perivascular space (i.e., the white area surrounding a vessel in Figure 5a) are clearly observed in $\tilde{I}$_HiLo_.

Due to the natural scattering properties of in vivo brain tissues, strong background noise is clearly observed in *I*_uni_ images. Compared to the above‐described ex vivo results of *I*_uni_ in Figure S18a (Supporting Information), the vessel inside the parenchymal brain, as shown in Figure 5a, is about three times thicker, and the background noise surrounding perivascular space is around twice high. However, with our RCNN model, $\tilde{I}$_HiLo_ demonstrates high‐contrast optical sectioning images, and background noise is significantly suppressed, which is evident from the zoom in results in Figure 5b. In all three depths, the model can successfully transfer the uniform input images into the high‐contrast optical sectioning images, which is equivalent to HiLo imaging results. Furthermore, the quantitative results of PSNR and SSIM in $\tilde{I}$_HiLo_ are 25.62 dB and 0.78, respectively, which is ≈12 dB and 5 times higher than those of *I*_uni_. It is important to note that even though in vivo mouse brain fluorescent images do not exist in the training dataset, the well‐trained RCNN model still can function equally to prove its generality. The tracer we inject into the mouse brain mainly flows into the perivascular space to show the glymphatic system, which causes the negatively visualized images.^[76^ ^]^ The additional results and discussion of in vivo fluorescence images of the left and right side of the mouse brain are shown in Section 15 of Supporting Information.

## 3 Conclusion

In summary, we demonstrate a high‐resolution optical sectioning meta‐device varifocal endo‐microscope, which utilizes a combination of compact varifocal metalens and an endoscopic probe in a telecentric design. A dielectric meta‐device is designed, fabricated, and characterized for this purpose. Due to the flat and compact nature of the metasurface, focus tunability is achieved with a miniature optical component with a thickness of less than 2 µm. The telecentric configuration of the meta‐device varifocal end‐microscopy provides uniform magnification throughout the axial scanning range. In addition, using a ray tracing matrix, we present a mathematical expression for focus tunability. The maximum focal length tuning range is ≈2 mm with 3 µm lateral resolution. Variable focus, together with the HiLo process, gives the advantage of non‐invasive multiplane imaging with fine optical sectioning.

The RCNN model is utilized to speed up imaging acquisition. The model is trained using various samples with different sizes and shapes. Results of the absolute error map show that predicted images using the RCNN have comparable optical sectioning capabilities as the conventional HiLo method. The well‐trained RCNN significantly suppresses the out‐of‐focus background noise and obtains high‐contrast fine features at the in‐focus plane. Compared to *I*_uni_, PSNR and SSIM values in the $\tilde{I}$_HiLo_ are enhanced to ≈14 dB and ≈4.5 times, respectively. The results show our RCNN model outperforms the basic U‐net model, and HiLo imaging computational time reduces from 5 s to 0.1 s, which makes our method conducive for high‐speed 3D imaging. With the help of the shortcut connection operations, the model turns into the counterpart residual version of inputs. Compared to the conventional U‐net, this capability positions RCNN as a pioneering architecture for capturing intricate hierarchical features in the data, facilitating the learning of complex patterns, and enhancing model performance. It solves vanishing/exploding gradients and degradation issues of the conventional convolutional neural network. The versatility and effectiveness of our trained RCNN model have been shown by predicting in vivo mouse brain vessels and surrounding perivascular space images, which do not exist in the training and validation datasets.

Focus tunability with optical sectioning potentially augments the diagnostic yield of existing endo‐microscopic imaging. By proper choice of fluorescent marker for near‐infrared imaging, penetration depth can be significantly extended. Compared to the conventional two‐photon technique employed for in vivo imaging, the proposed approach can offer unparalleled advantages such as large FOV, high imaging speed, and low cost‐effective. The direct impact of our volumetric endo‐microscopic imaging approach will be in performing rapid optical biopsies, which are required for various surgical procedures, including gastrointestinal biopsies, neural imaging, brain surgery, etc.

## 4 Experimental Section

### The Design and Fabrication of Moiré Metalens

The commercial numerical analysis tool Computer Simulation Technology Microwave Studio (CST MWS) for full‐wave simulation was applied, which was able to design and select the constituted meta‐atom for the metalens.^[^ ^71^, ^77^ ^]^ In the simulation process, the boundary conditions of cylinder meta‐atoms in the *x* and *y* directions was chosen, and both were unit cell modes, and the *z* direction was the open mode to optimize the simulation. The material of the dielectric substrate and meta‐atoms was sapphire (Al_2_O_3_, refractive index = 1.77 at λ = 532 nm) and Gallium Nitride (GaN, refractive index = 2.42 at λ = 532 nm), respectively as depicted in Figure S1a (Supporting Information). The height (H) of each GaN nano cylinder was 800 nm, and the period (P) of Al_2_O_3_ substrate was 300 nm. For the simulated spectral response (Figure S1b, Supporting Information), only the parameter setting (diameter, D) of cylinder meta‐atoms was picked with transmission efficiency higher than 80% to ensure high transmission performance of the metalens. Figure S1b (Supporting Information) shows the relationship of the meta‐atom size and the corresponding phase. Finally, using cylinder meta‐atoms (Table S1, Supporting Information) with different diameters, phase structures of Moiré metalens were designed.

For the fabrication procedure of the GaN Moiré metalens, the previous method was followed, as shown in Figure S2 (Supporting Information).^[^ ^35^, ^63^ ^]^ First, the 800 nm high refractive index GaN as the upper layer was grown on a low refractive index double‐polished Al_2_O_3_ substrate by the metalorganic chemical vapor deposition (MOCVD). And a 200 nm SiO_2_ thin film as a hard mask layer was deposited on the GaN layer by using an electron‐gun evaporator. Next, the photoresist (PMMA A4) was coated onto the SiO_2_ layer by spin coaters. After the substrate was prepared, the electron beam lithography system (Elionix ELS‐HS50) was utilized with 1 nA beam current and 50 kV acceleration voltage for the exposure procedure, transferring the unit cells arrangement of Moiré metalens to the photoresist layer. Then the substrate was immersed into the developer solution (MIBK: IPA = 1:3), and the 40 nm chromium layer was deposited onto the developed substrate by an electron‐gun evaporator to form the mask for the etching process. To remove the unnecessary photoresist on the surface, the substrate was soaked into the acetone solution for the lift‐off procedure. For the etching process, it can be divided into two steps. In the first step, the 90 W plasma power reactive‐ion etching (RIE) was utilized to transfer the designed pattern to the SiO2 mask layer. Second, the Cl_2_‐based inductively coupled plasma RIE (ICP‐RIE, 13.56 MHZ operated radio frequency) to etch the 800 nm GaN layer was used. Finally, the designed varifocal Moiré metalens can be generated by using a buffered oxide etch (BOE) solution to remove the unnecessary SiO_2_ layer.

### RCNN Model Implementation

The entire architecture of the proposed RCNN model was developed on the open‐source library TensorFlow (Version 2.4.0), and the model was trained and tested on a server‐level computer with 256 GB of RAM, two 2.4 GHz CPU cores (Xeon Silver 4210R, Intel Inc.), and dual RTX A5000 GPUs (24GB GPU Memory, NVIDIA Inc.). With the help of the NVIDIA CUDA GPU‐accelerated library (cuDNN), the entire training process was completed in ≈14.5 hours, which was 80 times faster than the non‐acceleration condition. In addition, the training optimizer for the RCNN model was a gradient decent‐based Adam optimization algorithm with the learning rate 1e‐3. The total training iteration (epochs) number was set to 3500, and the loss function for the model was the mean absolute error (MAE).

### Fixed Brain Imaging

Thirty minutes after the start of intracisternal injection, the mice have been sacrificed, and the brains were fixed overnight with 4% cold paraformaldehyde in PBS. To evaluating the tissue clearing images, the 250 µm brain coronal sections were cut on a vibratome (MicroSlicer DTK‐1000N, DSK, Japan), and tissue clearing was performed by RapiClear 1.47 (SunJin Lab Inc.), as described in the manufacturer's protocol.

### Intracisternal Injection for In Vivo Brain Imaging

The intracisternal injection method was based on previous literature with minor modifications.^[78^ ^]^ Mice (20≈25 g) were anesthetized intraperitoneally with a combination of Zoletil (50 mg k^−1^g) and xylazine (2.3 mg k^−1^g). After confirming the loss of response to a toe pinch, mice were placed in a stereotaxic frame (Stoelting, USA), and the cisterna magna was exposed through a surgical incision. A 30‐gauge needle connected to a Hamilton syringe via a polyethylene tube (PE10) was implanted in the cisterna magna, and the catheter was secured via cyanoacrylate glue and dental cement. 10 µl CSF tracer (5 mg ml^−1^ albumin‐Alexa Fluor 555, ThermoFisher Scientific, Cat no. A34786, USA) was infused at a constant rate of 1 µl min^−1^ with a syringe pump (Harvard Apparatus, USA). Twenty minutes later, the tracer was observed transcranially through fluorescence microscopy (Olympus, Cat No. MVX10, Japan) to confirm that the tracer has entered the perivascular space, and then the skull was opened and the in vivo brain imaging was ready for the proposed endo‐microscopy.

### Animals

All animal experiments were carried out at the National Taiwan University College of Medicine in accordance with the guidelines of Institutional Animal Care and Use Committee (IACUC approval No. 20 201 028, 20 220 503 & 20 230 043). Mice (C57BL/6JNarl) have access to food and water ad libitum and were maintained in a climate‐controlled room under a 12 h/12 h of light/dark cycle.

## Conflict of Interest

The authors declare no conflict of interest.

## Author Contributions

Y.H.C. and W.H.L. contributed equally to this work. Y.H.C., C.H.C., and S.V. conceived the design, and performed the numerical design, optical measurement, and data analysis. W.H.L. and W.S.C. performed the in vivo and ex vivo sample preparation. C.H.C., T.Y., M.K.C., T.T., Y.L. and D.P.T. conceived the principle, design, and characterization of the metasurface and meta‐optics of the system. Y.H.C., X.L., and M.K.C. built up the optical system for measurement and the deep learning model and collected the dataset for the model. Y.H.C., S.V., Y.L., Y.Y.H., W.S.C., and D.P.T. organized the project, designed experiments, analyzed the results, and prepared the manuscripts. All authors discussed the results and commented on the manuscript.

## Supporting information

Supporting Information

## Data Availability

The data that support the findings of this study are available from the corresponding author upon reasonable request.
