# Supplementary material for: In Vivo Intelligent Fluorescence Endo‐Microscopy by Varifocal Meta‐Device and Deep Learning
Source: Adv Sci (Weinh). 2024 Mar 15;11(20):2307837. doi: 10.1002/advs.202307837 (PMC11132035; doi:10.1002/advs.202307837)
Supplement: Supplementary file 1 — Supporting Information [file ADVS-11-2307837-s001.pdf]

ADVANCED SCIENCE Open Access

Supporting Information

for *Adv. Sci.*, DOI 10.1002/advs.202307837

In Vivo Intelligent Fluorescence Endo-Microscopy by Varifocal Meta-Device and Deep Learning

Yu-Hsin Chia, Wei-Hao Liao, Sunil Vyas, Cheng Hung Chu, Takeshi Yamaguchi, Xiaoyuan Liu, Takuo Tanaka, Yi-You Huang, Mu Ku Chen, Wen-Shiang Chen*, Din Ping Tsai* and Yuan Luo**

Supplementary Materials for

In-vivo intelligent fluorescence endo-microscopy by varifocal meta-device and deep learning

Yu-Hsin Chia[1,2†], Wei-Hao Liao[3†], Sunil Vyas[2], Cheng Hung Chu[4], Takeshi Yamaguchi[5], Xiaoyuan Liu[6], Takuo Tanaka[5], Yi-You Huang[1,2,7], Mu Ku Chen[6,8,9]*, Wen-Shiang Chen[3,10]*, Din Ping Tsai[6,8,9]*, Yuan Luo[2,4,11,12]*

[1]Department of Biomedical Engineering, National Taiwan University, Taipei, 10051, Taiwan

[2]Institute of Medical Device and Imaging, National Taiwan University, Taipei, 10051, Taiwan

[3]Department of Physical Medicine and Rehabilitation, National Taiwan University Hospital & National Taiwan University College of Medicine, Taipei, 10051, Taiwan

[4]YongLin Institute of Health, National Taiwan University, Taipei, 10087, Taiwan

[5]Innovative Photon Manipulation Research Team, RIKEN Center for Advanced Photonics, Saitama 351-0198, Japan

[6]Department of Electrical Engineering, City University of Hong Kong, Kowloon 999077, Hong Kong

[7]Department of Biomedical Engineering, National Taiwan University Hospital, Taipei, 10051, Taiwan

[8]Centre for Biosystems, Neuroscience and Nanotechnology, City University of Hong Kong, Kowloon, 999077, Hong Kong

[9]The State Key Laboratory of Terahertz and Millimeter Waves, City University of Hong Kong, Kowloon, 999077, Hong Kong

[10]Institute of Biomedical Engineering and Nanomedicine, National Health Research Institutes, Miaoli, Taiwan

[11]Molecular Imaging Center, National Taiwan University, Taipei, 10672, Taiwan

[12]Program for Precision Health and Intelligent Medicine, National Taiwan University, Taipei, 106319, Taiwan

†These authors contributed equally to this work.

*Corresponding author: mkchen@cityu.edu.hk, wenshiang@gmail.com, dptsai@cityu.edu.hk, yuanluo@ntu.edu.tw

This file includes:

Section 1: The design of GaN meta-atom for metasurface

Section 2: Moiré metalens fabrication procedure

Section 3: Telecentric configuration of the meta-varifocal endo-microscopy

Section 4: Ray transfer matrix for the telecentric design

Section 5: The telecentric focusing measurement

Section 6: HiLo imaging principle

Section 7: Varifocal optical sectioning endo-microscopy calibration

Section 8: *Ex-vivo* mouse brain imaging results

Section 9: RCNN DL model for optical sectioning endo-microscopy

Section 10: Original U-net model architecture

Section 11: Residual convolutional neural network (RCNN) model architecture

Section 12: Prediction results of RCNN model validation dataset

Section 13: Prediction results of RCNN model training dataset

Section 14: Quantitative evaluation metrics for model

Section 15: *Ex-vivo* mouse brain imaging prediction from the RCNN model

Section 16: *In-vivo* imaging prediction from the RCNN model

Section 1: The design of GaN meta-atom for metasurface

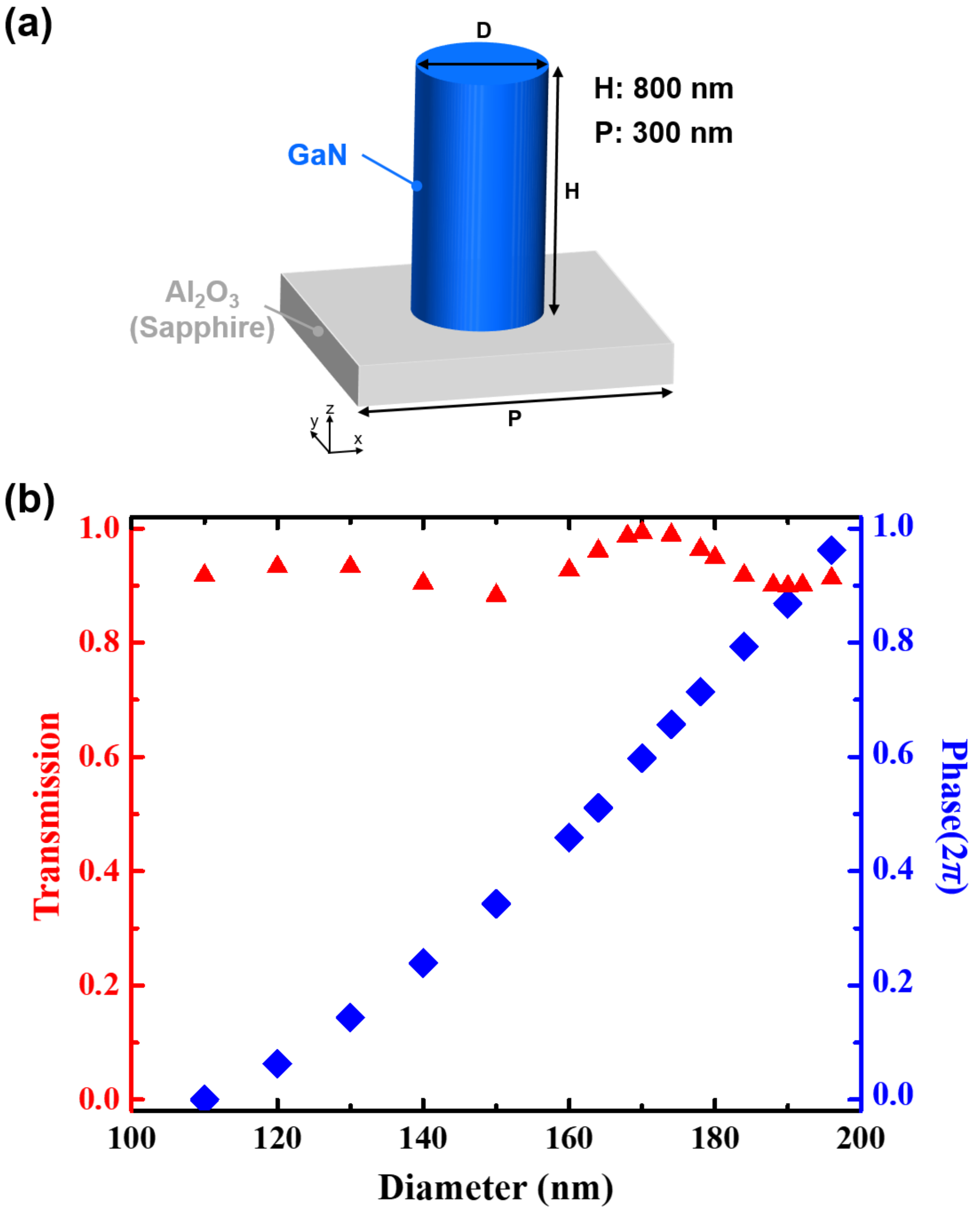

Fig. S1. GaN meta-atom specification. (a) Meta-atom for Moiré metalens consisting of GaN nano cylinder and Al_2O_3 sapphire substrate with 800 nm height (H) and 300 nm period (P). (b) Simulated results of transmission and phase spectra with different meta-atom diameters.

Table S1. Transmission and phase shift of the cylinder meta-atoms with different diameter.

Diameter (D, nm)	Transmission	Phase (°)
110	0.918	0
120	0.933	22.69
130	0.933	51.66
140	0.904	86.08
150	0.883	123.42
160	0.927	165.27
164	0.961	184.08
168	0.987	204.6
170	0.993	215.18
174	0.989	236.55
178	0.964	257.15
180	0.949	266.87
184	0.918	285.69
188	0.901	303.52
190	0.899	312.57
192	0.901	159.39
196	0.913	346.47

Section 2: Moiré metalens fabrication procedure

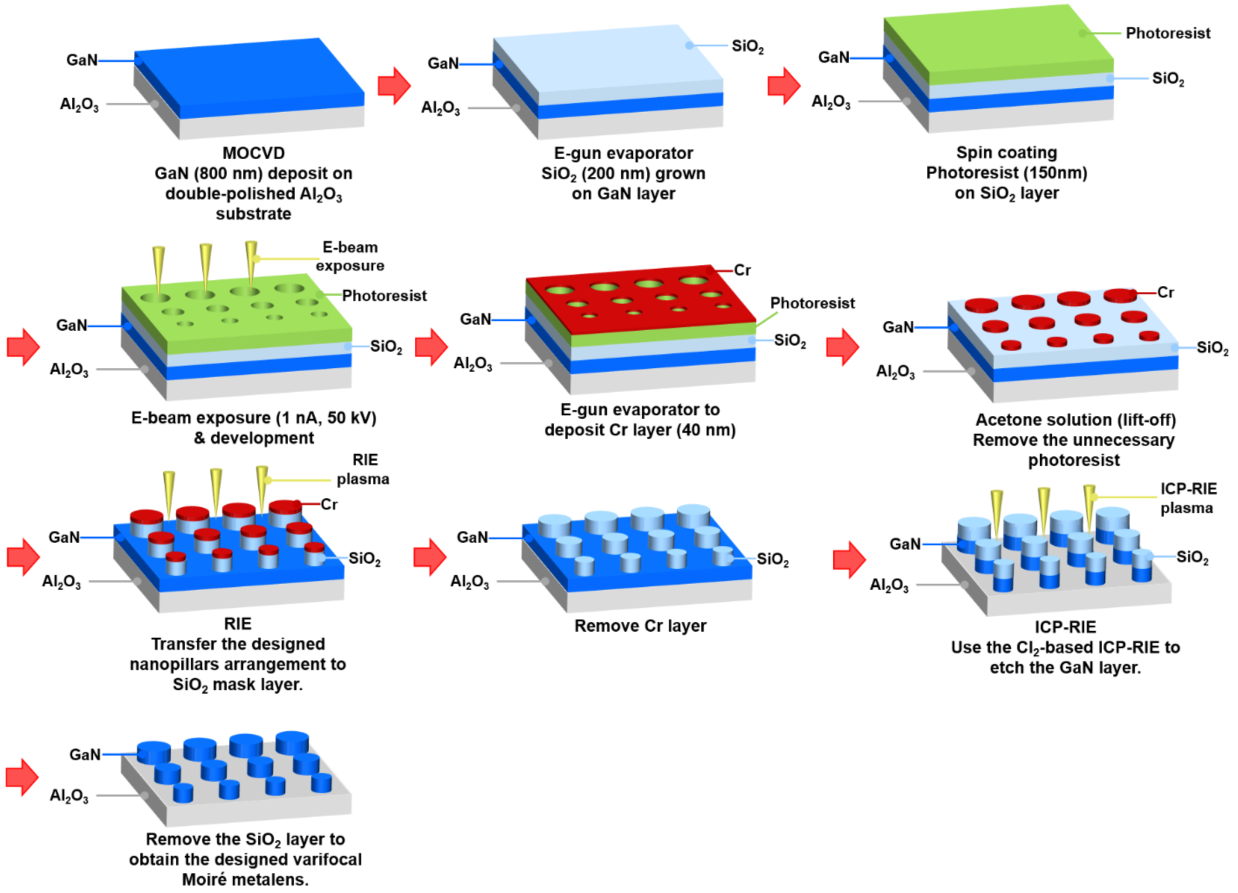

Fig. S2. Fabrication procedure of the GaN Moiré metalens.

Section 3: Telecentric configuration of the meta-varifocal endo-microscopy

Figure S3 shows the experimental setup and measurement of telecentric configuration for the endo-microscope. Figure S3 (a) shows the telecentric configuration for our endo-microscope. The measurements of the endoscope's associated focal length and NA at various Moiré metalens rotation angles are depicted in Figure S3 (b). To verify the invariant magnification property of our endo-microscope, a resolution chart is placed at the different axial focal planes of the endoscope probe depicted in Figure S3 (c). The resolution chart is shifted from the initial in focus plane (z = 0 μm) to z = 1500 μm plane with 300 μm spacing, which changes from sharp images to blurred images (top row of Figure S3 (c)). By rotating the Moiré metalens, with relative angles from 5° to 120°, the corresponding defocused image can be refocused to in-focus (bottom row of Figure S3 (c)). The results show that the telecentric design keeps both magnification and FOV as constant.

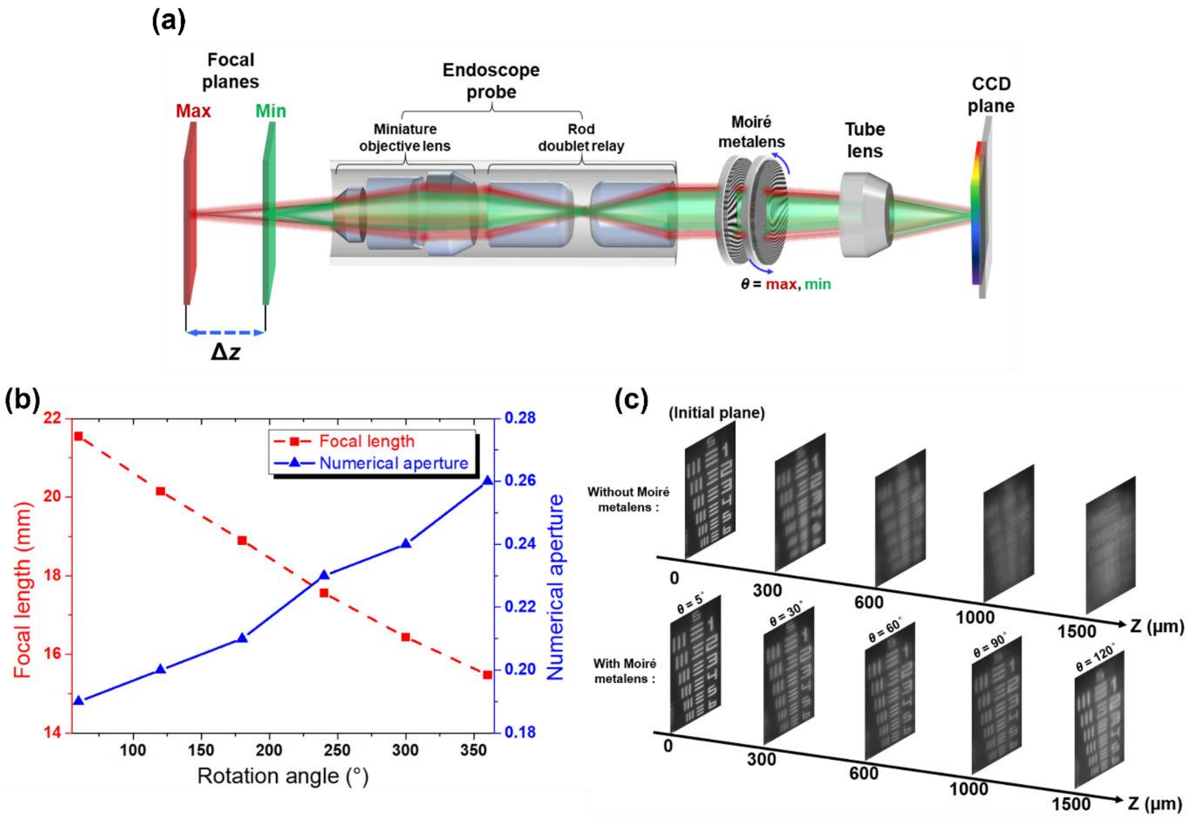

Fig. S3. Experimental setup and measurement of the Moiré metalens based varifocal endo-microscopy. (a) The telecentric setup of endo-microscopy, which includes a miniature objective lens, rod achromatic doublet relay, Moiré metalens, and tube lens. (b) Experimental results of focal length and the corresponding NA of the endoscope at different Moiré metalens rotation angles. (c) The digital axial scanning for multi-plane images.

Section 4: Ray transfer matrix for the telecentric design

Ray transfer matrix calculation can help us confirm the constant magnification of the telecentric design(*1*). In Figure S4, the focal length of the endoscope probe is f_E. The 4-f relay lens (R1 and R2) consists of focal lengths with f_{R1} and f_{R2}, and the lengths of the Moiré metalens and tube lenses are f_{Moire} and f_T, respectively. Ray transfer matrix for propagation (P) in a medium with a constant refractive index is given by

$$P = \begin{bmatrix} 1 & d \\ 0 & 1 \end{bmatrix}, \quad \text{(S1)}$$

where d is the separation distance between two reference surfaces along the optical axis. We assume that the optical lens components used in Figure S4 are all thin lenses, which can follow lens transfer matrix (L)

$$L = \begin{bmatrix} 1 & 0 \\ -\frac{1}{f} & 1 \end{bmatrix}, \tag{S2}$$

where f is the focal length of the optical lens. The ray transfer matrix of Moiré metalens based varifocal endo-microscopy (M) can be determined as

$$M = P_T \times L_T \times P_{T-Moire} \times L_{Moire} \times P_{Moire-R_2} \times L_{R_2} \times P_{R_2-R_1} \times L_{R_1} \times P_{R_1-E} \times L_E , \tag{S3}$$

where the $P_{T\text{-}Moire}$ is corresponding to the air propagation matrix from the Moiré metalens to the tube lens. We can divide the entire transfer matrix M into several parts that include endoscope probe matrix (M_E), 4-f relay lens matrix (M_R), Moiré metalens matrix (M_{Moire}) and tube lens matrix (M_T). These yield the following equations

$$M_E = \begin{bmatrix} 1 & 0 \\ -\frac{1}{f_E} & 1 \end{bmatrix}, \tag{S4}$$

$$M_R = \begin{bmatrix} -\frac{f_{R2}}{f_{R1}} & f_{R1} - \frac{f_{R2} f_E}{f_{R1}} \\ 0 & -\frac{f_{R1}}{f_{R2}} \end{bmatrix}, \tag{S5}$$

$$M_{Moire} = \begin{bmatrix} 1 - \frac{f_T}{f_{Moire}} & f_{R2} - \frac{f_T f_{R2}}{f_{Moire}} + f_T \\ -\frac{1}{f_{Moire}} & -\frac{f_{R2}}{f_{Moire}} + 1 \end{bmatrix}, \tag{S6}$$

$$M_T = \begin{bmatrix} 0 & f_T \\ -\frac{1}{f_T} & 1 \end{bmatrix}. \tag{S7}$$

After multiplying all the matrices, the entire matrix can be written as

$$M = \begin{bmatrix} \frac{f_{R1} f_T}{f_E f_{R2}} & \frac{f_T f_{R2}^2 f_E - f_{R1}^2 f_{Moire} f_T}{f_{R1} f_{R2} f_{Moire}} \\ 0 & \frac{f_{R2} f_E}{f_{R1} f_T} \end{bmatrix}. \tag{S8}$$

From the ray transfer matrix, magnification of the entire system can be described in the first term of the matrix ($M_{1,1}$), which indicates that the system magnification does not depend on the focal length of the Moiré metalens (f_{Moire}) to satisfy the telecentric design. In addition, the axial position of the focusing beam (F) can be computed as

$$F = -\frac{M_{1,2}}{M_{1,1}} = f_E - \frac{{f_{R2}}^2 {f_E}^2}{{f_{R1}}^2 f_{Moire}}. \tag{S9}$$

The total axial displacement of the focusing beam (Δz) in telecentric design can be calculated as

$$\begin{aligned}\Delta z = F_{\mathrm{Max}} - F_{\mathrm{Min}} &= \frac{{f_{R2}}^2 {f_E}^2}{{f_{R1}}^2 f_{Moire,\mathrm{Min}}} - \frac{{f_{R2}}^2 {f_E}^2}{{f_{R1}}^2 f_{Moire,\mathrm{Max}}} \\ &= \frac{{f_{R2}}^2 {f_E}^2}{{f_{R1}}^2}\left(\frac{1}{f_{Moire,\mathrm{Min}}} - \frac{1}{f_{Moire,\mathrm{Max}}}\right), \\ &= \frac{{f_{R2}}^2\, {f_E}^2\, \lambda\, c}{{f_{R1}}^2\, \pi}\left(\theta_{\mathrm{Max}} - \theta_{\mathrm{Min}}\right)\end{aligned} \tag{S10}$$

where F_{Max}, F_{Min} are the maximum and minimum axial position of the focusing beam, which can be given by $f_{Moire,\mathrm{Min}}$ and $f_{Moire,\mathrm{Max}}$, respectively.

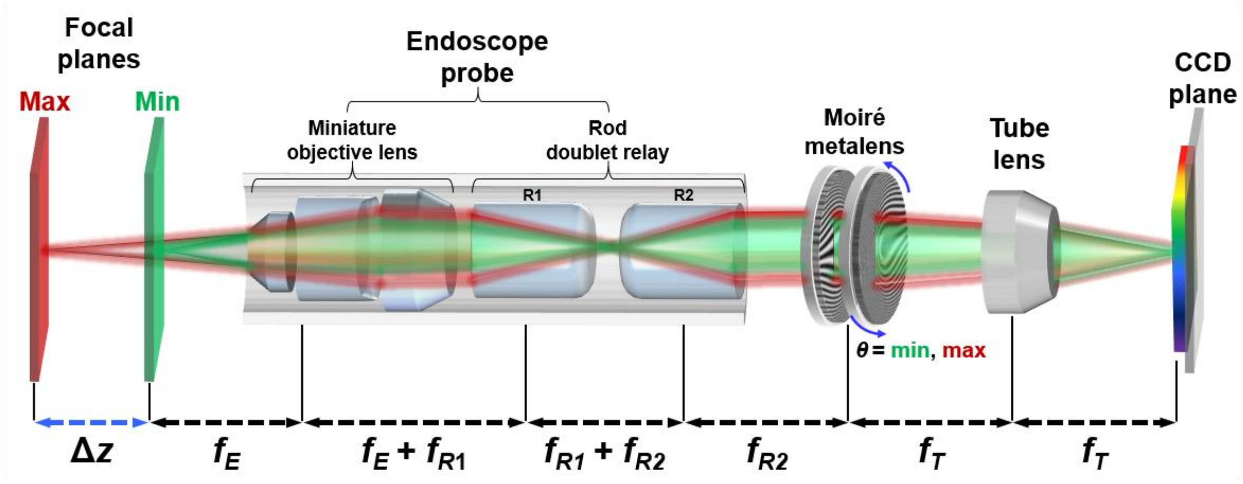

Fig. S4. The telecentric configuration of the Moiré metalens based varifocal endo-microscopy.

Section 5: The telecentric focusing measurement

In telecentric focusing measurement results, we make the laser beam source go through a focus lens to generate the point source beam at the image plane. The point source pass through the tube lens to become the parallel beam to illuminate moiré metalens. After the beam pass through the endoscope probe can generate the focus point at focal plane. The corresponding focal length of the endoscope probe can be tuned when the rotation angles of the moiré metalens are adjusted. Finally, we set a CCD on a motorized linear stage to execute the axial scanning to measure telecentric focusing measurement results as shown in Figure S5. Except for green wavelength, we also measure the telecentric tuning distance under the blue (491 nm) and red (633 nm) wavelengths, as shown in Figures S6 and S7. With the blue laser, the focal length of the endoscope probe can be tuned from ~ 15.4 to ~ 20.6 mm (Δz = 5.15 mm) at Moiré metalens rotation angle of 360°to 60°. The NA of endoscope probe changes from ~ 0.26 to ~ 0.19. The lateral and axial resolutions vary from 1.15 to 1.54 μm and 18.84 to 33.51 μm, respectively. With the red laser, the focal length of the endoscope probe can be tuned from ~ 15.7 to ~ 23.4 mm (Δz = 7.68 mm) and the corresponding NA values vary from ~ 0.26 to ~ 0.17. The lateral and axial resolutions vary from 1.51 to 2.25 μm and 19.5 to 43.25 μm, respectively. The experimental results show our system can be operated for broad visible spectrum from 491 nm to 633 nm.

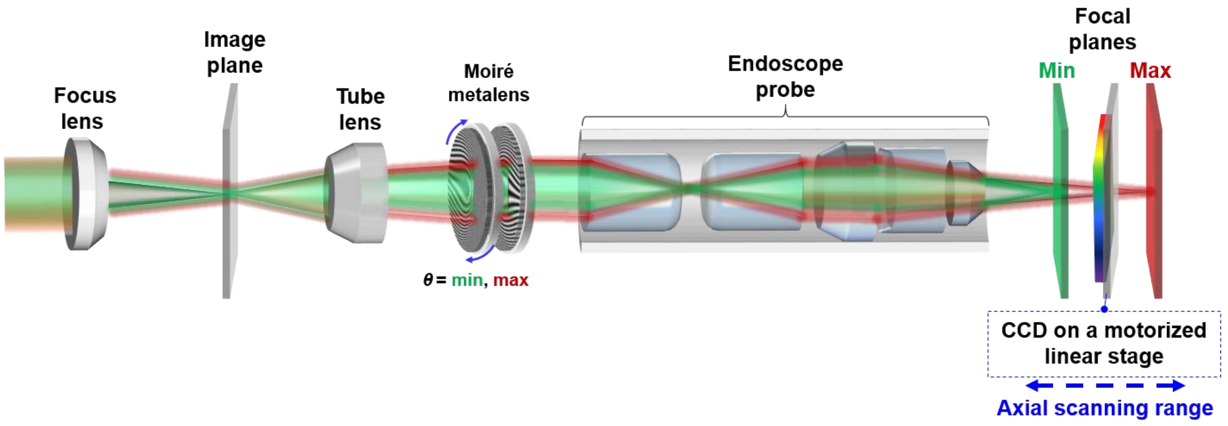

Fig. S5. Telecentric focusing measurement setup.

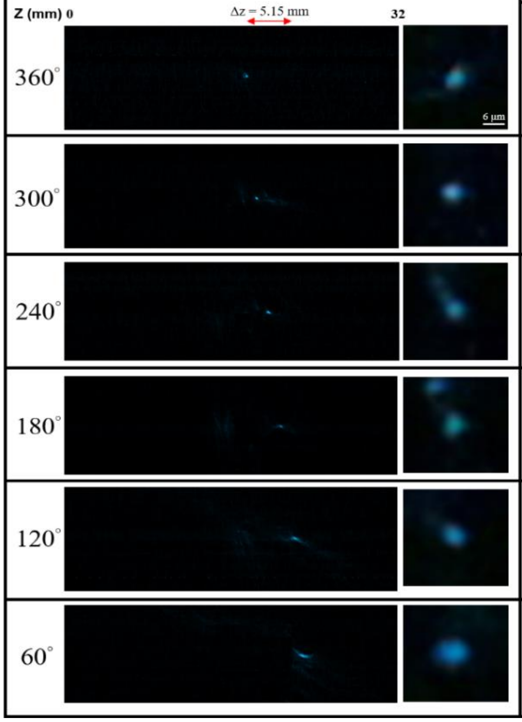

	Focal length	NA	Lateral resolution	Axial resolution
360°	15.43mm	0.26	1.15 μm	18.84 μm
300°	16.32mm	0.26	1.22 μm	21.07 μm
240°	17.36mm	0.23	1.29 μm	23.85 μm
180°	18.41mm	0.22	1.38 μm	26.82 μm
120°	19.67mm	0.20	1.47 μm	30.61 μm
60°	20.58mm	0.19	1.54 μm	33.51 μm

Fig. S6. Telecentric focusing behavior measurement of blue wavelength at 491 nm.

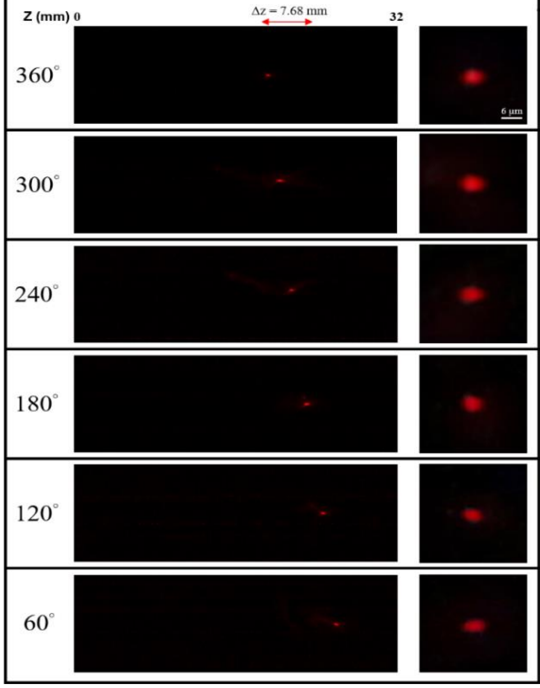

	Focal length	NA	Lateral resolution	Axial resolution
360°	15.70mm	0.26	1.51 μm	19.50 μm
300°	17.05mm	0.24	1.65 μm	23.00 μm
240°	18.39mm	0.22	1.78 μm	26.76 μm
180°	20.03mm	0.20	1.93 μm	31.74 μm
120°	21.78mm	0.18	2.10 μm	37.53 μm
60°	23.38mm	0.17	2.25 μm	43.25 μm

Fig. S7. Telecentric focusing behavior measurement of red wavelength at 633 nm.

Section 6: HiLo imaging principle

HiLo imaging process is a very efficient method to obtain optical sectioning images in wide-field microscopy (*2*-*5*). For one HiLo optically sectioned image, it requires a pair of images in distinct illumination situation: one is under the uniform illumination (I_{uni}), and the other is under speckle illumination (I_{sp}). I_{uni} can be represented as the combination of the in-focus component (I_{inf}) and defocus component (I_{def})

$$I_{uni}(x,y) = I_{inf}(x,y) + I_{def}(x,y) , \tag{S11}$$

where *x*, *y* represents the spatial coordinates at two-dimensional image plane.

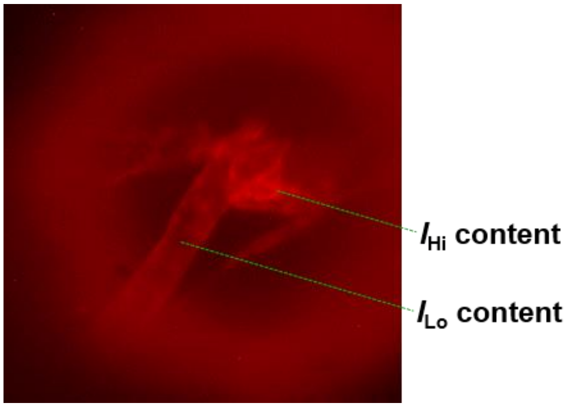

Fig. S8. Uniform illumination image (I_{uni}) of mouse brain perivascular spaces.

Figure S8 shows the I_{uni} image of mouse brain perivascular spaces. The high spatial frequencies content (I_{Hi}) of the HiLo optical sectioned image can be directly extract from the I_{uni}

$$I_{Hi}(x,y) = I_{uni}(x,y) * GHP_{cut} , \tag{S12}$$

where GHP_{cut} is the two dimensional Gaussian high-pass filter with certain cut-off frequency (*cut*). On the other hand, the speckle illumination image (I_{sp}) can then be decomposed as

$$I_{sp}(x,y) = I_{inf}(x,y)M(x,y) + I_{def}(x,y) , \tag{S13}$$

where the $M(x,y)$ is the modulation coefficient of the speckle illumination depicted in Figure S9.

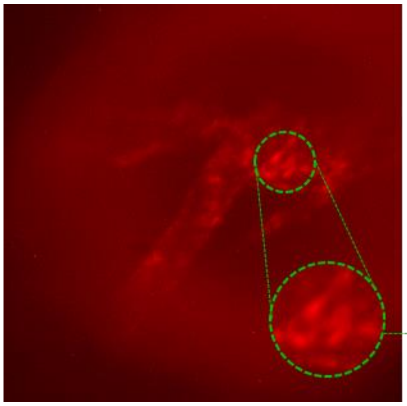

Fig. S9. Speckle illumination image (I_{sp}).

In-focus area of the speckle illumination images has the highest contrast, while the defocus area has low values. Therefore, the low spatial frequencies content (I_{Lo}) of the HiLo image can be written as

$$I_{Lo}(x,y) = [I_{uni}(x,y) \times LSC(x,y)] * GLP_{cut}, \tag{S14}$$

where GLP_{cut} is the two-dimensional Gaussian low-pass filter that has the identical cut-off frequency with GHP_{cut}. $LSC(x,y)$ is the local spatial contrast of the I_{sp}, it can be calculated as

$$LSC(x,y) = \frac{< STD(I_{sp}) >_{sw}}{< MV(I_{sp}) >_{sw}}, \tag{S15}$$

where the $<MV(I_{sp})>_{sw}$ and $<STD(I_{sp})>_{sw}$ represent the mean value and standard deviation of the I_{sp}, which are computed with the sampling window (sw). Hence, the multiplication of $I_{uni}(x,y)$ and $LSC(x,y)$ is able to eliminate the defocus component and extract the in-focus component. Finally, Figure S10 shows the HiLo optical sectioned image ($I_{HiLo}(x,y)$). $I_{HiLo}(x,y)$ is generated by merging both high spatial frequencies content (I_{Hi}) andlow spatial frequencies content (I_{Lo}) to obtain the in-focus image with the entire spatial frequency range, and it can be expressed as

$$I_{HiLo}(x,y) = I_{Hi}(x,y) + [sf \times I_{Lo}(x,y)]. \tag{S16}$$

The scaling factor (sf, typical values is the range 0.5 ~ 3) is able to balance the intensity distribution between the $I_{Hi}(x,y)$ and $I_{Lo}(x,y)$. Here, in our case the sf = 1 to obtain high-contrast image result.

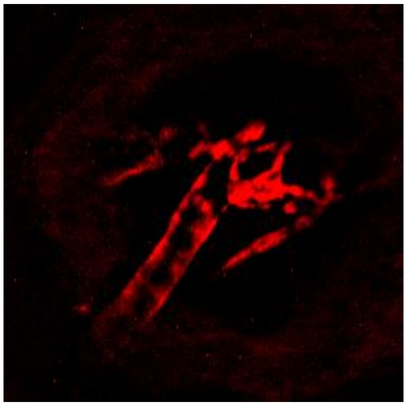

Fig. S10. HiLo optical sectioned image (I_{HiLo}).

Section 7: Varifocal optical sectioning endo-microscopy calibration

By rotating the angle between paired metasurfaces, the microspheres are imaged with a step size (Δz) of 0.3 μm and their normalized intensity profiles are shown in Figure S11 (a). Intensity variation at different depths is calculated at the same region of interest (ROI), and gray values inside the ROI are averaged to measure sectioning capability. In Figure S11 (a), the full width at half maximum (FWHM) under uniform illumination is ~ 100 μm, and the FWHM of HiLo process is ~ 35 μm, which shows direct and solid evidence that HiLo imaging method empowers optical sectioning capability.

To verify optical sectioning capability for biomedical tissue, *ex-vivo* fluorescently labeled transparent mouse brains are imaged by the micro-endoscope. The water-soluble clearing reagent (RapiClear ® 1.49, SunJin Lab Inc.) is utilized in the cleaning process, which makes the *ex-vivo* mouse brain tissue transparent and clear for observing fine structures (the detailed preparation process for fixed brain imaging is discussed in Materials and Methods). We adopt fluorescent tracers that conjugate with Alexa Fluor™ 488 and Alexa Fluor™ 555 to observe the diffusion of CSF in the brain via cisterna magna injection, and the tracers are able to influx into the perivascular space. Detailed structures, labeled with Alexa Fluor™ 555 (central emission λ ~ 600 nm), are imaged using the green laser for excitation (image results of detailed structures, labeled with Alexa Fluor™ 488, are shown in Section 8 of Supplementary Materials). The *ex-vivo* images (I_{uni}) of the transparent brain tissue with a thickness of ~ 250 μm under uniform illumination at two different depths are shown in Figure S11 (b). By adjusting the angles of the Moiré metalens from 5°to 20°, the focus plane is tuned with the range of 200 μm. Due to the inherent scattering effect within the volumetric brain tissue, out-of-focus haze background can be obviously observed under uniform

illumination. With HiLo imaging, the strong haze background can be significantly reduced to obtain optically sectioned images. The fine structure around perivascular spaces is clearly imaged in I_{HiLo}, as shown in bottom row of Figure S11 (b).

The 3D optical sectioning capability of our endo-microscopy is also evaluated by sequentially imaging *ex-vivo* transparent brain tissue, along the axial direction, with a step size (Δz) of 10 µm. Figure S11 (c) shows 3D images of various perivascular space locations with a volume size of 750 µm × 750 µm × 1 mm. In 3D I_{uni} images, strong background noise caused by scattering severely degrades image quality, and thus the detailed structures of the brain tissue are hardly to be resolved. Compared to I_{uni} images, the out-of-focus background noise is significantly suppressed in I_{HiLo} images, which provide 3D optical sectioning images with a high signal-to-noise ratio. Our endo-microscopy demonstrates the ability to resolve clear detailed structures of *ex-vivo* mouse brain tissue, such as the vessels deep in the perivascular space. According to the specification of our electrically controlled rotation stage (GT45, Dima Inc.), the maximum rotation speed is 140 RPM = 2.3 rev/second. Moreover, the max frame rate of our CCD (GE1650, Prosilica Inc.) is 32 fps. The fundamental requirement of real-time imaging is about 20 fps, therefore based on the specification of the rotation stage and CCD, our endoscopic system has high potential to satisfy the 3D real-time imaging.

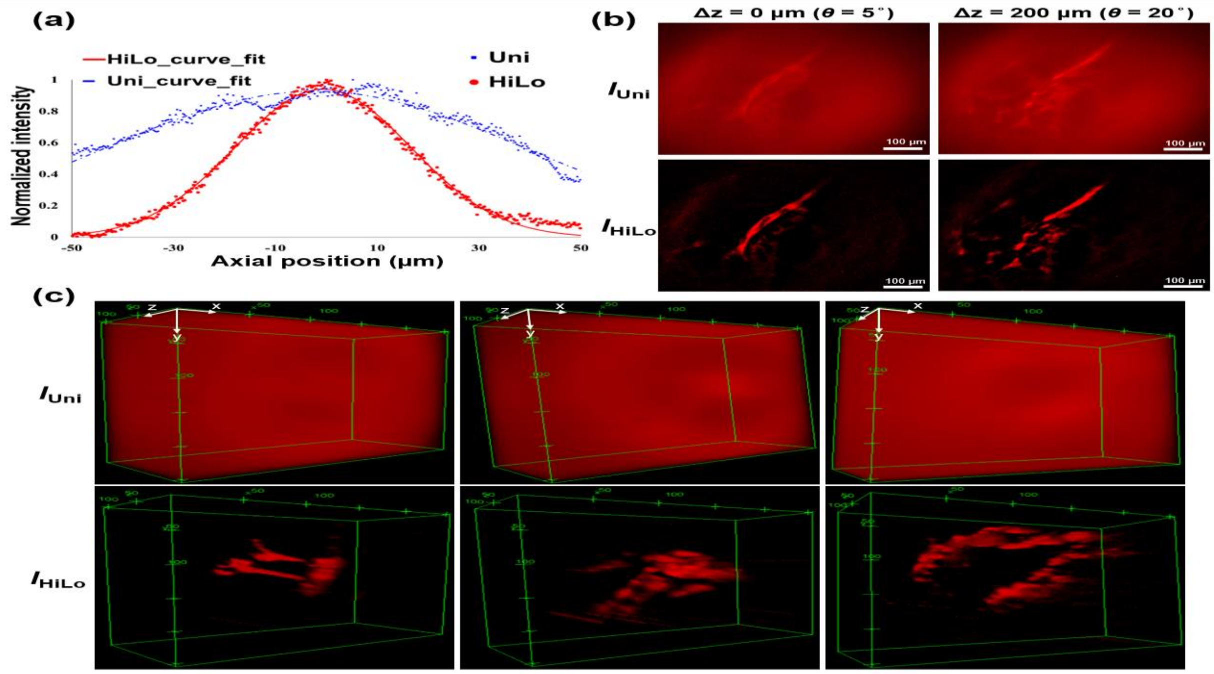

Fig. S11. Varifocal optical sectioning endo-microscopy calibration. (a) Normalized intensity distribution of fluorescent microspheres along the axial direction. Blue and red data points are measured using uniform illumination and HiLo imaging process, respectively (dots: measured raw data, curve: Gaussian curve fitting). **(b)** Two different depths of *ex-vivo* transparent mouse brain tissue (250 μm thickness) tagged with Alexa Fluor™ 555 in both wide-field (*i.e.* I_{uni}) and HiLo (I_{HiLo}) imaging results. **(c)** 3D reconstruction volume images of mouse brain various perivascular space locations in uniform illumination and HiLo process (each 3D image is 750 μm × 750 μm × 1 mm).

Section 8: *Ex-vivo* mouse brain imaging results

For the brain tissue dyed with Alexa Fluor 488, a blue laser operated at 491 nm (Cobolt Calypso 200) is used to excite the green fluorescence, with central wavelength of 525 nm. *Ex-vivo* images of the transparent mouse brain tissue with the thickness of 250 μm under uniform illumination for two different depths are shown in Figure S12 (a). Two images, separated with distance of 200 μm in depths, are obtained by tuning mutual angles of the two metasurfaces at 5° and 20°, respectively. Figure S12 (b) shows the corresponding HiLo optical sectioning images. From the comparison of intensity cross-sections in Figure S12 (c), high-contrast optically sectioned images are obtained by the HiLo method.

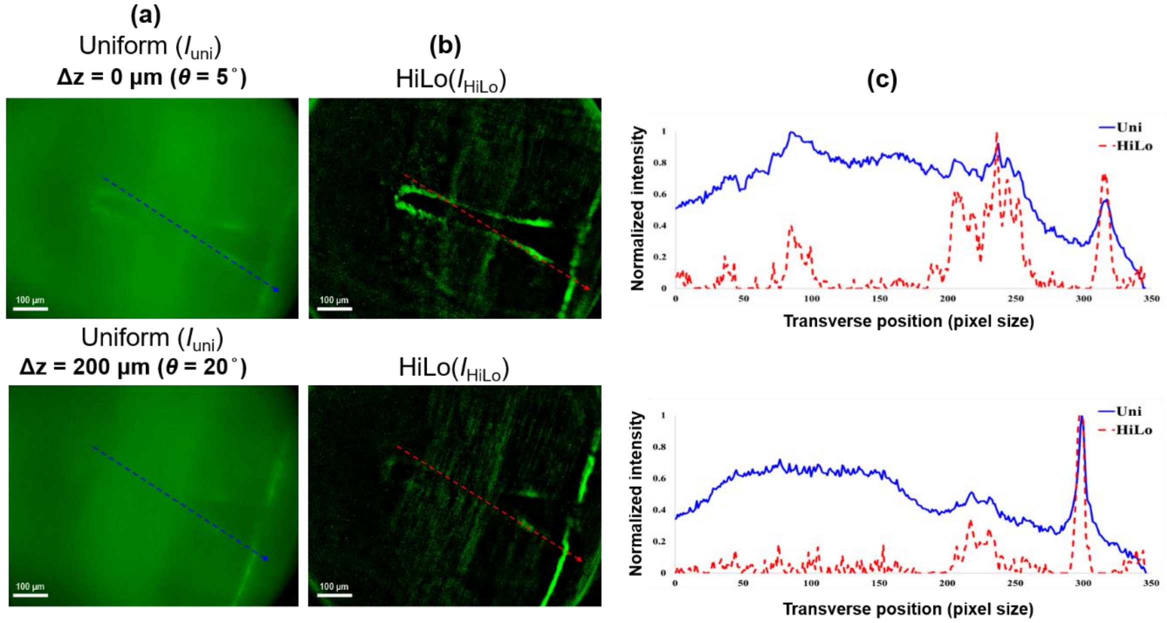

Fig. S12. Comparison results of Alexa Fluor 488 labeled *ex-vivo* transparent mouse brain. (a) Wide-field images, (b) HiLo optical sectioning images, and (c) comparison of normalized intensity profile.

We have performed *ex-vivo* image of the thicker transparent mouse brain tissue (500 µm thickness) dyed with Alexa Fluor 488 as shown in Figure S13. Compared with the uniform illumination image (Figure S13 (a)), the HiLo imaging (Figure S13 (b)) significantly suppresses out-of-focus background noise. Figure S13 (c) shows comparison of the intensity profile of central hollow structures of brain tissue indicated by the dashed lines.

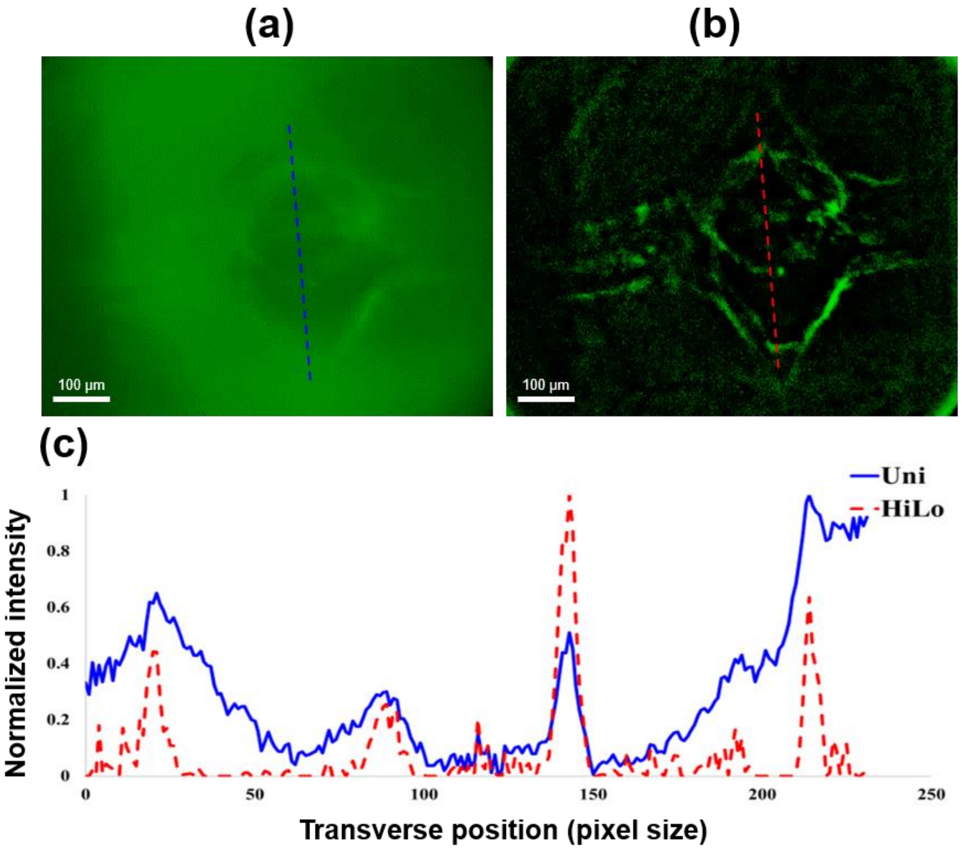

Fig. S13. Comparison results of Alexa Fluor 488 labeled *ex-vivo* transparent mouse brain tissue. (a) Wide-field images, (b) HiLo optical sectioning images, and (c) comparison of normalized intensity profile.

Section 9: RCNN DL model for optical sectioning endo-microscopy

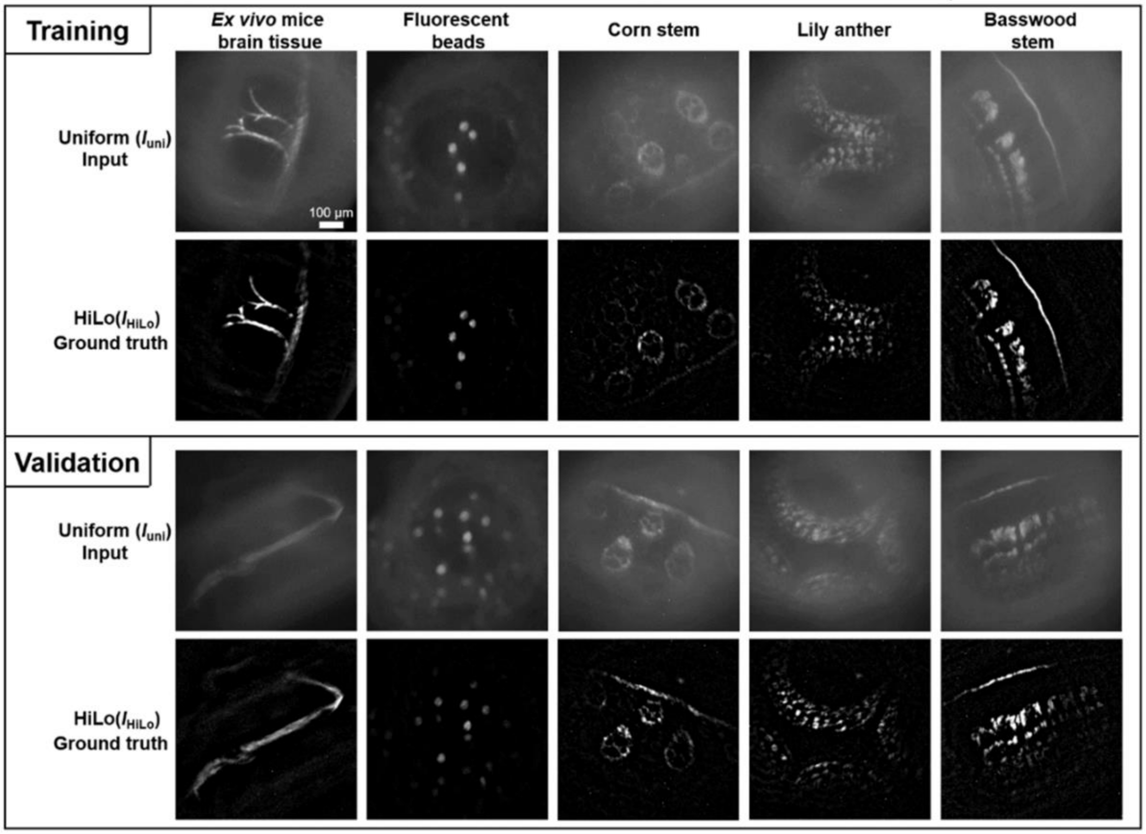

Fig. S14. Randomly selected data of five different types of fluorescent samples with 2800 training and 700 validation dataset image pairs.

Section 10: Original U-net model architecture

Figure S15 demonstrates the architecture of the original U-net model. The original U-Net model for HiLo optical sectioning images includes 19 convolutional layers. Both the size of the input and output image are 256×256 pixels. In the encoder part, 10 convolutional layers and 4 max pooling layers are used to make the input image can gradually down-sampling from 256×256 to 16×16 pixels. The kernel size in the encoder of each convolutional layer is set to 4×4, and the activation function of each convolutional layer is chosen as a leaky rectified linear unit (LReLU) with a slope of 0.2. In the decoder path, 9 convolutional layers (4×4 kernel size) and 4 transpose convolution layers (blue arrows in Figure S15) are utilized to up-sampling and regress the image from 16×16 to 256×256 pixels. The activation function for decoder in each convolutional layer is rectified

linear unit (ReLU). For the last layer, the activation function is applied the tanh. Between the encoder and decoder, the skip connection is used to concatenate the features that match between the down-sampling and up-sampling processes.

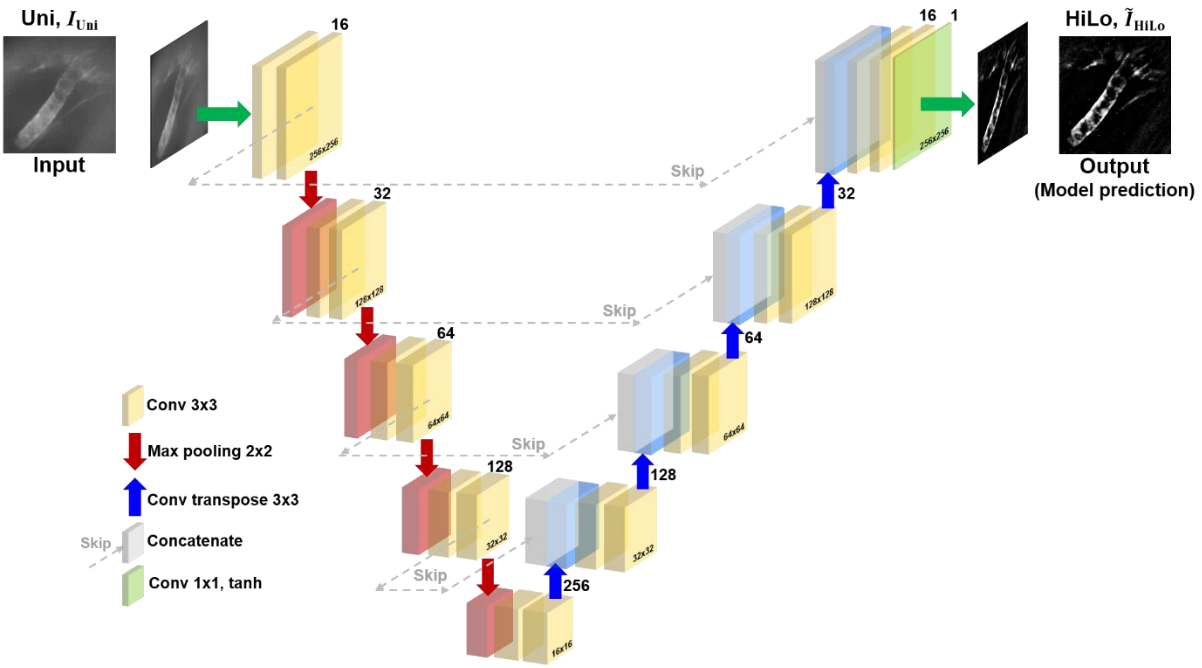

Fig. S15. Original U-net model for HiLo optical sectioning images.

Section 11: Residual convolutional neural network (RCNN) model architecture

Figure S16 shows the structure of the RCNN model to obtain optical sectioning images from the wide-field. Both input and output images are 256×256 pixels. The RCNN model is based on the U-net architecture that includes the encoder and decoder part(*6*, *7*). In the encoder, 4 residual down-sampling blocks are used to make the input image gradually down-sampled from 256×256 to 16×16 pixels to generate different size of feature maps. Each residual down-sampling block includes two convolutional layers (yellow square), shortcut connections (black arrow) and max pooling layer (red arrow) depicted in Figure S17 (a). In the decoder path, 4 residual up-sampling blocks are utilized to up-sampling and regress the image from 16×16 to 256×256 pixels. One residual up-sampling block consists of two convolutional layers (yellow square), shortcut connections (black arrow) and up-sampling layer (blue arrow) depicted in Figure S17 (b). Detailed parameters of each layer for the encoder and decoder are listed in Tables S2 and S3, respectively.

The kernel size for each convolutional layer is set to 3×3 with stride (1,1) and the leaky rectified linear unit (LReLu) with slope 0.5 is chosen to be the corresponding activation function. The shortcut connection is operated by utilizing the identity shortcuts that use 3×3 convolutional filters with stride (1,1) to make the input and output have the same dimensions to add together. For the last layer, the tanh activation function is applied. Between the encoder and decoder, the skip connection is used to concatenate the features that match between the down-sampling and up-sampling processes. This step can make the down-sampling low level features directly pass to the high level layers in up-sampling, which is helpful for image transformation. In addition, the dropout layer and batch normalization layer are applied to the model, which can induce more stabilization in training process and performance. With the help of the shortcut connection operations, the model turns into the counterpart residual version of inputs. It solves vanishing/exploding gradients and degradation issue of the conventional convolutional neural network.

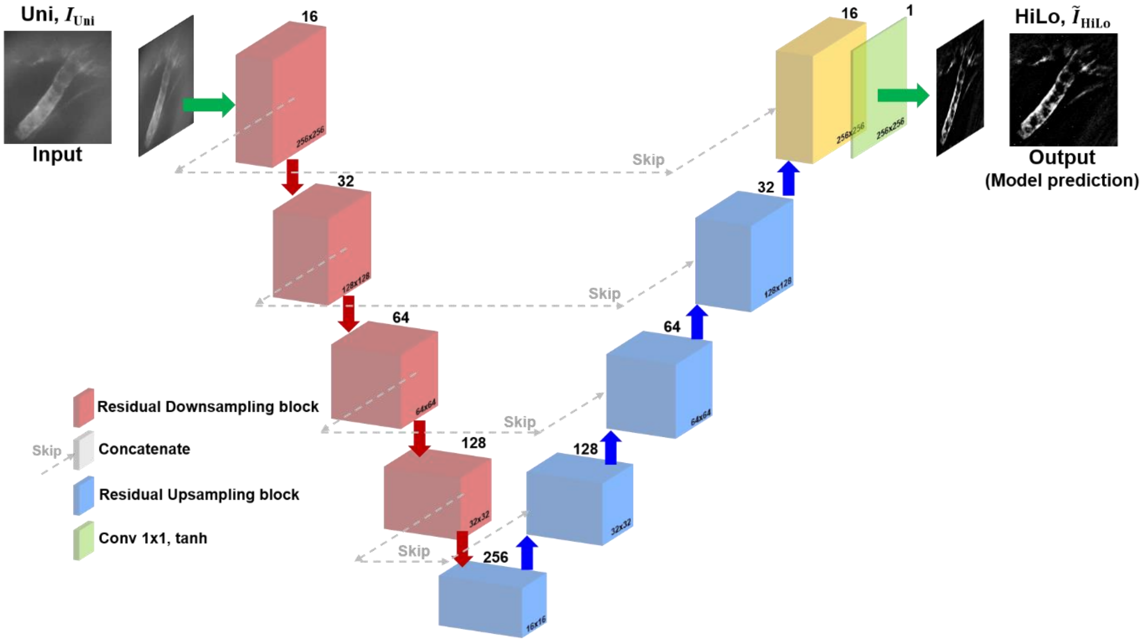

Fig. S16. RCNN model for HiLo optical sectioning images.

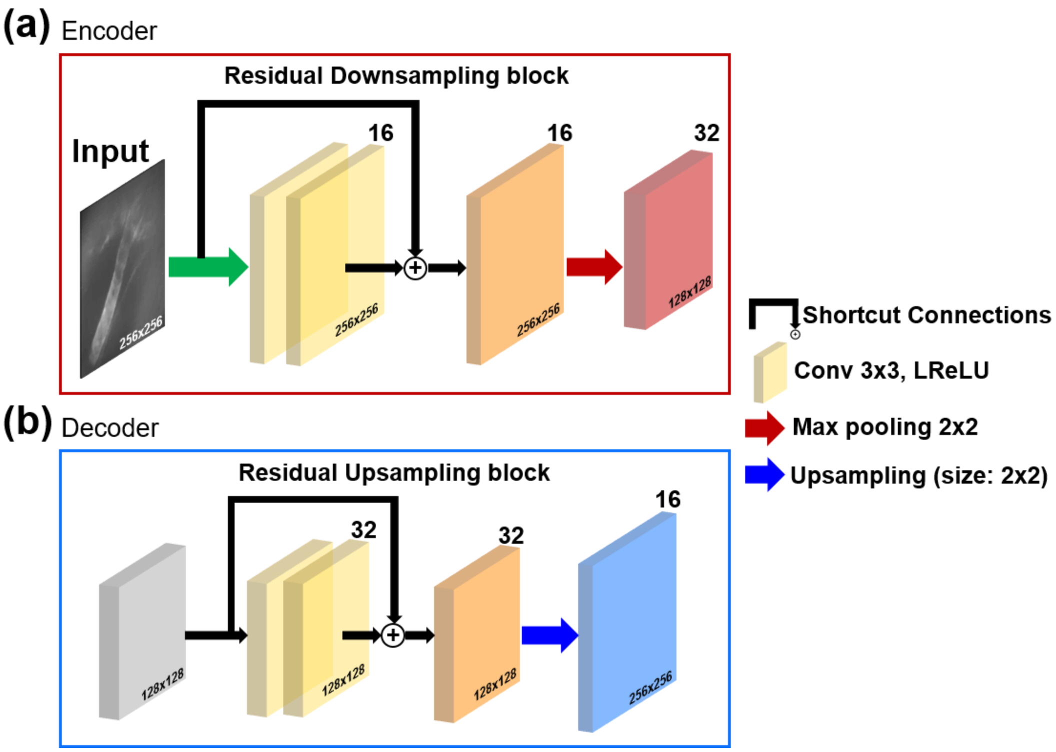

Fig. S17. Detailed architecture of the RCNN model. (a) Residual down-sampling block for encoder. (b) Residual up-sampling blocks for decoder.

Table S2. The encoder architecture of the RCNN model

Input	Output	Output shape	Type
Inputs	Inputs	(256,256,1)	Input image
Inputs	Conv1_1	(256,256,16)	Conv2D 3x3
Conv1_1	Conv1_2	(256,256,16)	Conv2D 3x3
Inputs	Shortcut_1	(256,256,16)	Conv2D 3x3
Conv1_2, Shortcut_1	Sum_1	(256,256,16)	Add
Sum_1	Pool1	(128,128,16)	MaxPooling2D
Pool1	Conv2_1	(128,128,32)	Conv2D 3x3
Conv2_1	Conv2_2	(128,128,32)	Conv2D 3x3
Pool1	Shortcut_2	(128,128,32)	Conv2D 3x3
Conv2_2, Shortcut_2	Sum_2	(128,128,32)	Add
Sum_2	Pool2	(64,64,32)	MaxPooling2D
Pool2	Conv3_1	(64,64,64)	Conv2D 3x3
Conv3_1	Conv3_2	(64,64,64)	Conv2D 3x3
Pool2	Shortcut_3	(64,64,64)	Conv2D 3x3
Conv3_2, Shortcut_3	Sum_3	(64,64,64)	Add
Sum_3	Pool3	(32,32,64)	MaxPooling2D
Pool3	Conv4_1	(32,32,128)	Conv2D 3x3
Conv4_1	Conv4_2	(32,32,128)	Conv2D 3x3
Pool3	Shortcut_4	(32,32,128)	Conv2D 3x3
Conv4_2, Shortcut_4	Sum_4	(32,32,128)	Add
Sum_4	Pool4	(16,16,128)	MaxPooling2D
Pool4	Conv5_1	(16,16,256)	Conv2D 3x3
Conv5_1	Conv5_2	(16,16,256)	Conv2D 3x3
Pool4	Shortcut_5	(16,16,256)	Conv2D 3x3
Conv5_2, Shortcut_5	Sum_5	(16,16,256)	Add

Table S3. The decoder architecture of the RCNN model

Input	Output	Output shape	Type
Sum_5	Up_1	(32,32,256)	UpSampling2D
Sum_4, Up_1	Merge_1	(32,32,384)	Concatenate
Merge_1	Conv6_1	(32,32,128)	Conv2D 3×3
Conv6_1	Conv6_2	(32,32,128)	Conv2D 3×3
Merge_1	Shortcut_6	(32,32,128)	Conv2D 3×3
Conv6_2, Shortcut_6	Sum_6	(32,32,128)	Add
Sum_6	Up_2	(64,64,128)	UpSampling2D
Sum_3, Up_2	Merge_2	(64,64,192)	Concatenate
Merge_2	Conv7_1	(64,64,64)	Conv2D 3×3
Conv7_1	Conv7_2	(64,64,64)	Conv2D 3×3
Merge_2	Shortcut_7	(64,64,64)	Conv2D 3×3
Conv7_2, Shortcut_7	Sum_7	(64,64,64)	Add
Sum_7	Up_3	(128,128,64)	UpSampling2D
Sum_2, Up_3	Merge_3	(128,128,96)	Concatenate
Merge_3	Conv8_1	(128,128,32)	Conv2D 3×3
Conv8_1	Conv8_2	(128,128,32)	Conv2D 3×3
Merge_3	Shortcut_8	(128,128,32)	Conv2D 3×3
Conv8_2, Shortcut_8	Sum_8	(128,128,32)	Add
Sum_8	Up_4	(256,256,32)	UpSampling2D
Sum_1, Up_4	Merge_4	(256,256,48)	Concatenate
Merge_4	Conv9_1	(256,256,16)	Conv2D 3×3
Conv9_1	Conv9_2	(256,256,16)	Conv2D 3×3
Merge_4	Shortcut_9	(256,256,16)	Conv2D 3×3
Conv9_2, Shortcut_9	Sum_9	(256,256,16)	Add
Sum_9	Conv10_1	(256,256,1)	Conv2D 1×1

Section 12: Prediction results of RCNN model validation dataset

The left column of Figure S18 is the absolute error between the I_{uni} and the I_{HiLo}, while the right column is the difference between the $\tilde{I}_{HiLo}$ and I_{HiLo}. Therefore, the predicted images ($\tilde{I}_{HiLo}$) shows optical sectioning images, which is comparable to the ground truth (I_{HiLo}). The absolute error maps demonstrate that the trained RCNN model significantly suppresses out-of-focus background noise and enhances signal-to-noise ratio of in-focus plane images.

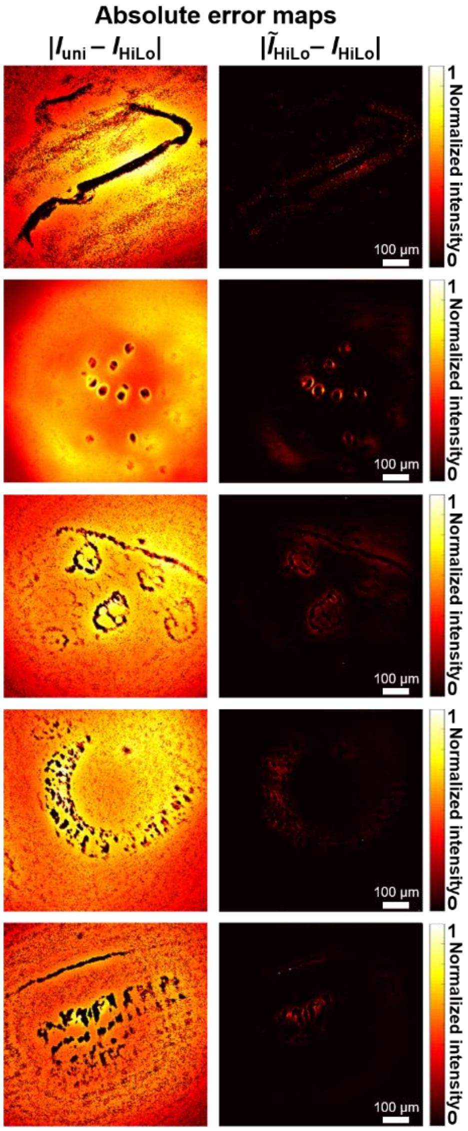

Fig. S18. Comparison of inputs, ground truths, and predictions from validation dataset. Absolute error maps on the left column show difference between the input and the ground truth

images. Absolute error maps on the right column show the difference between the prediction and ground truth images.

Section 13: Prediction results of RCNN model training dataset

To test the generality of trained RCNN model, we compared the input, ground truth, and prediction images from the training dataset depicted in Figure S19, which shows similar results as the validation dataset (Figure S18). Our model removes the background noise and increases the signal-to-noise ratio of the predicted images.

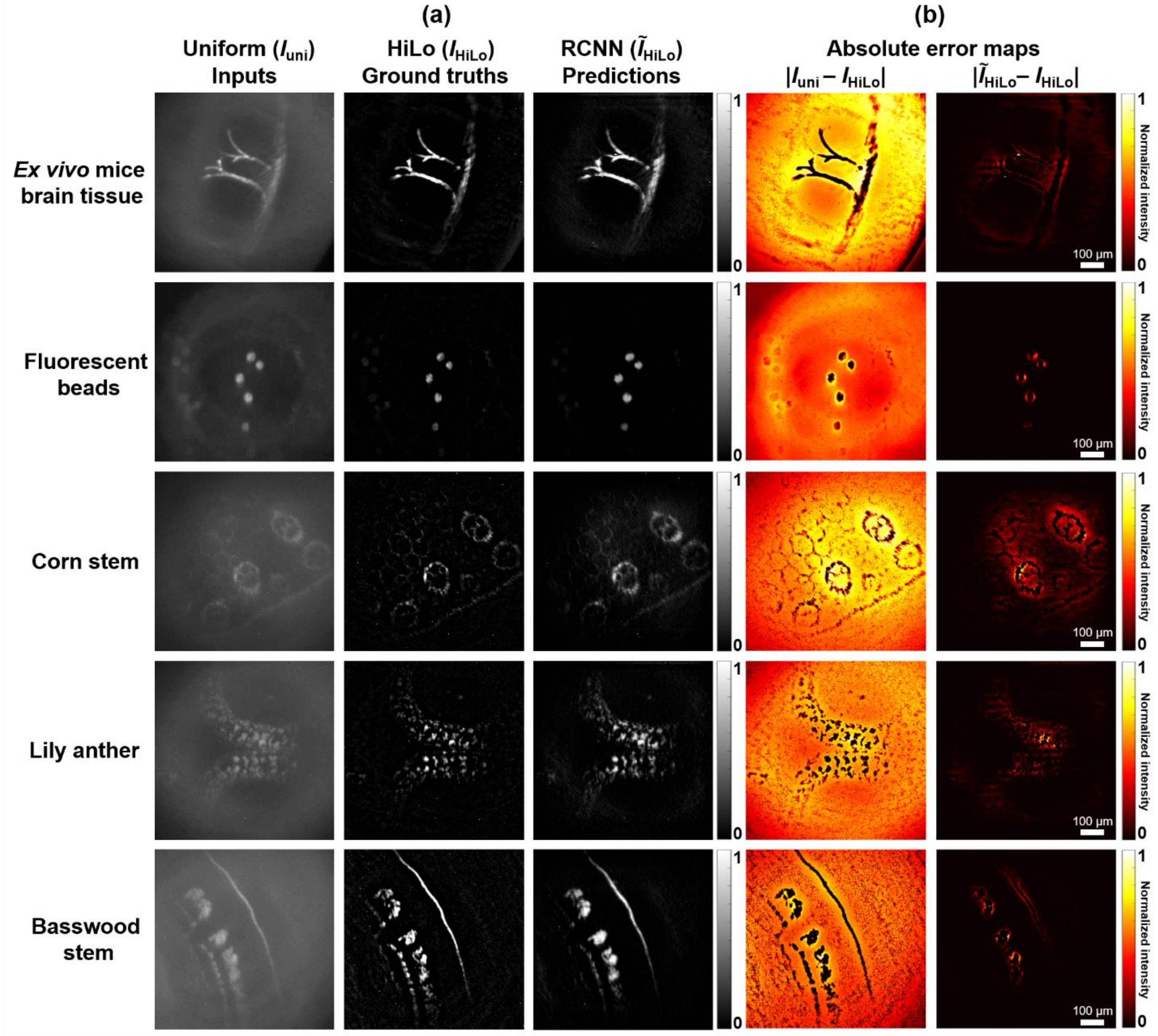

Fig. S19. The comparison results of the inputs, ground truths, and predictions from the training dataset. (a) Resultant images of five different types of fluorescent samples that include *ex-vivo* mouse brain tissue, fluorescent beads, corn stem, lily anther, and basswood stem taken by

training dataset. (b) Left column of absolute error maps are the difference between the input and the ground truth images. Right column of absolute error maps is the difference between the prediction and ground truth images.

Section 14: Quantitative evaluation metrics for model

The performance of the RCNN model can be quantitatively accessed by two different kinds of evaluation metrics(*8*). First is the peak signal to noise ratio (PSNR), which can indicate the quality of the images to quantify the model's reconstruction quality. PSNR is defined by the difference between two images and it can be computed as follows

$$PSNR = 20 \cdot \log_{10}\left(\frac{MAX_I}{\sqrt{MSE}}\right), \tag{S17}$$

where the MAX_I means the maximum value of the image. MSE is the mean squared error that can be written as

$$MSE = \sum_{x=1}^{X}\sum_{y=1}^{Y}\left(I_{\mathrm{m}}(x,y) - I_{\mathrm{HiLo}}(x,y)\right)^2, \tag{S18}$$

where I_{m} is the input (I_{uni}) or model predict images ($\tilde{I}_{\mathrm{HiLo}}$). The X and Y represent the width and height of one sectioning image, and x and y are the spatial pixel coordinates in each sectioning image. If the PSNR is higher, it means that the predicted images by the RCNN model have comparable image quality with the ground truth images (I_{HiLo}).

Structural similarity (SSIM) is the second evaluation metric, and it can quantify the similarity between the model prediction and ground truth images. SSIM can be calculated as

$$SSIM\left(I_{\mathrm{m1}}, I_{\mathrm{m2}}\right) = [lu\left(I_{\mathrm{m1}}, I_{\mathrm{m2}}\right)^{\alpha} \cdot cn\left(I_{\mathrm{m1}}, I_{\mathrm{m2}}\right)^{\beta} \cdot st\left(I_{\mathrm{m1}}, I_{\mathrm{m2}}\right)^{\gamma}], \tag{S19}$$

where the I_{m1} and I_{m2} are the two images to be compared. The I_{m1} represents the input or model predict image and I_{m2} is the ground truth images. The *lu*, *cn,* and *st* are the luminance, contrast, and structure, respectively. α, β, and γ mean the weighting factors for corresponding parameters, and here we set it to unity.

Table S4 shows PSNR and SSIM values for input and predicted images from the validation dataset. Compared to the input images, the average PSNR and SSIM values from the RCNN predicted images is improved ~ 14 dB and 4.5 times, respectively. From Table S4, both PSNR and

SSIM in the RCNN model offer the highest values, which demonstrates that our RCNN has better performance and advantages than the conventional U-net due to shortcut connections.

Table S4. Quantitative comparison of the input and DL predicted images

	PSNR	SSIM
**Input with ground truth**	18.60 dB	0.18
**U-net with ground truth**	25.94 dB	0.66
**RCNN with ground truth**	32.27 dB	0.81

Section 15: *Ex-vivo* mouse brain imaging prediction from the RCNN model

Figure S20 (a) shows *ex-vivo* images of I_{uni}, I_{HiLo} and $\tilde{I}_{\mathrm{HiLo}}$, at different depths. Due to the lack of transparent process, the brain tissue generates much more scattered background noise signal, making the fluorescent tracer inside the brain tissue hard to observe clearly under the uniform illumination. With the well-trained RCNN model, the wide-field images (*i.e.* I_{uni}) can directly transfer into corresponding optical sectioning images (*i.e.* $\tilde{I}_{\mathrm{HiLo}}$). In Figure S20 (a), fine features of perivascular space are clearly observed. The absolute error maps in Figure S20 (b) demonstrate that predicted $\tilde{I}_{\mathrm{HiLo}}$ have comparable optical sectioning images with the HiLo images (*i.e.* I_{HiLo}). The predicted average PSNR and SSIM metrics of *ex-vivo* brain images at three different depths are 31.61 dB and 0.82, which match the validation dataset values.

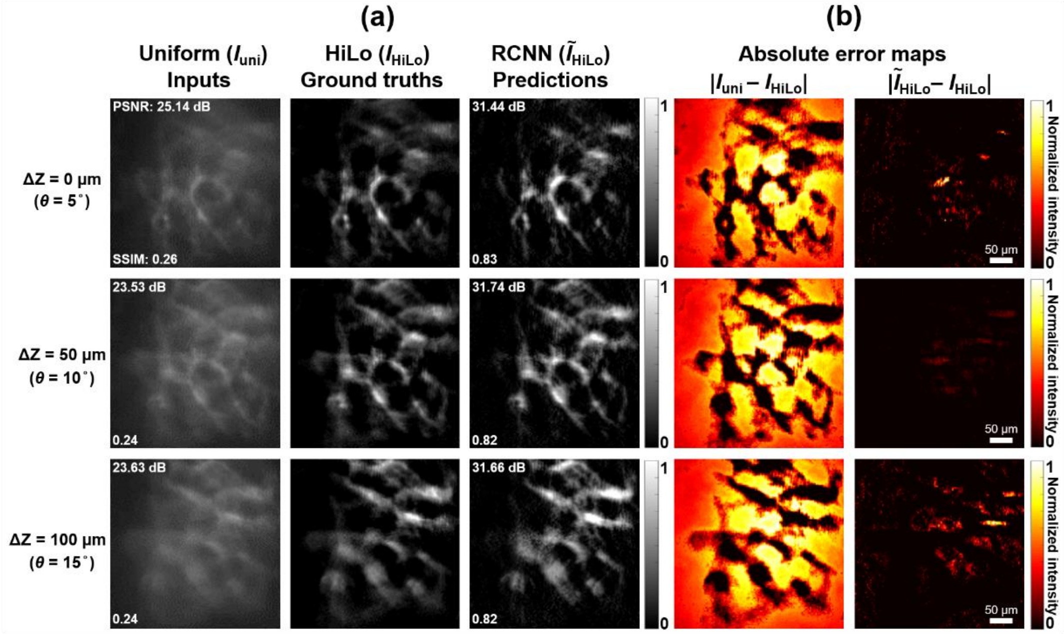

Fig. S20. Images comparison results for the *ex-vivo* mouse brain. a, With corresponding Moiré metalens rotation angles, I_{uni}, I_{HiLo} and $\tilde{I}_{\mathrm{HiLo}}$ fluorescent images at three different depths. **b,** Absolute error maps in the left column show the difference between I_{uni} and I_{HiLo}, while absolute error maps in the right column show the difference between $\tilde{I}_{\mathrm{HiLo}}$ and I_{HiLo}.

Section 16: *In-vivo* imaging prediction from the RCNN model

To demonstrate the well-trained RCNN model for the *in-vivo* preclinical applications, we have taken high-contrast optical sectioning images of both right and left side of *in-vivo* mouse brain, as shown in Figure S21 and S22. With our RCNN model, $\tilde{I}_{\mathrm{HiLo}}$ demonstrate high-contrast optical sectioning images, and background noise is significantly suppressed, which is evident from the absolute error maps in Figure S21 (b) and S22 (b).

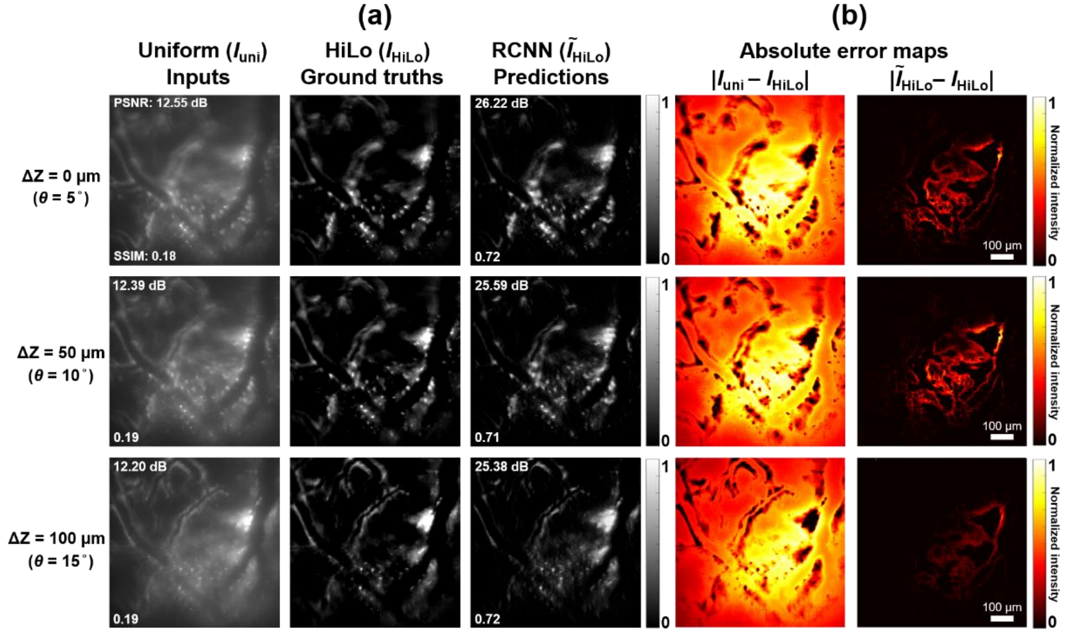

Fig. S21. *In-vivo* fluorescence images of right side of mouse brain. a, *In-vivo* images of wide-field, ground truth, and model predictions at three different depths. **b,** Absolute error maps comparison results on the left column show the difference between I_{uni} and I_{HiLo}, while absolute error maps on the right column show the difference between $\tilde{I}_{\mathrm{HiLo}}$ and I_{HiLo}.

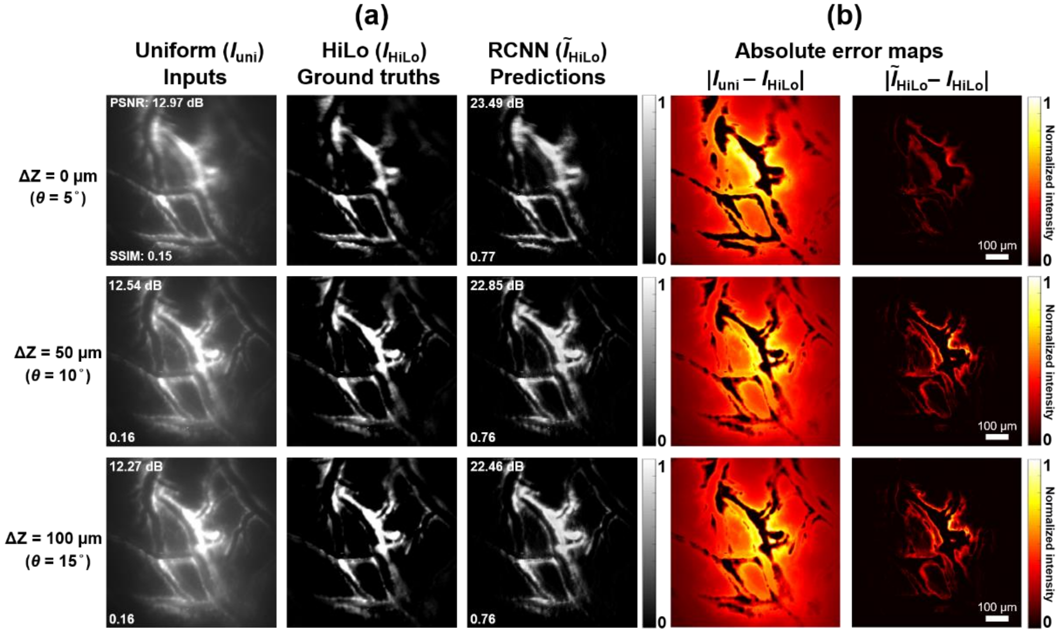

Fig. S22. ***In-vivo* fluorescence images of left side of mouse brain. a,** *In-vivo* images of wide-field, ground truth, and model predictions at three different depths. **b,** Absolute error maps comparison results on the left column show the difference between I_{uni} and I_{HiLo}, while absolute error maps on the right column show the difference between $\tilde{I}_{\text{HiLo}}$ and I_{HiLo}.

References

1. G. Sancataldo *et al.*, Three-dimensional multiple-particle tracking with nanometric precision over tunable axial ranges. *Optica* **4**, 367-373 (2017).
2. D. Lim, K. K. Chu, J. Mertz, Wide-field fluorescence sectioning with hybrid speckle and uniform-illumination microscopy. *Opt. Lett.* **33**, 1819-1821 (2008).
3. D. Lim, T. N. Ford, K. K. Chu, J. Metz, Optically sectioned in vivo imaging with speckle illumination HiLo microscopy. *J. Biomed. Opt.* **16**, 016014 (2011).
4. J. Mertz, Optical sectioning microscopy with planar or structured illumination. *Nat. Methods.* **8**, 811-819 (2011).
5. T. N. Ford, D. Lim, J. Mertz, Fast optically sectioned fluorescence HiLo endomicroscopy. *J. Biomed. Opt.* **17**, 021105 (2012).
6. A. Sinha, J. Lee, S. Li, G. Barbastathis, Lensless computational imaging through deep learning. *Optica* **4**, 1117-1125 (2017).
7. K. He, X. Zhang, S. Ren, J. Sun, Deep residual learning for image recognition. *IEEE conference on computer vision and pattern recognition*, 770-778 (2016).
8. A. Hore, D. Ziou, Image quality metrics: PSNR vs. SSIM. *2010 20th international conference on pattern recognition. IEEE*, 2366-2369 (2010).
